# Engineered multifunctional nanoparticles for enhanced radiation therapy: three-in-one approach for cancer treatment

**DOI:** 10.1186/s12943-025-02266-1

**Published:** 2025-03-06

**Authors:** Tejaswini Appidi, Debarghya China, George-Răzvan Ștefan, Michele Moreau, Serena Mao, Esteban Velarde, Ngeh Toyang, Henry Lowe, Aravind Kumar Rengan, Kai Ding, Wilfred Ngwa

**Affiliations:** 1 Department of Radiation Oncology and Molecular Radiation Sciences, Johns Hopkins University School of Medicine, Baltimore, Maryland USA; 2 Department of Biomedical Engineering, Johns Hopkins University, Baltimore, Maryland USA; 3 Carol Davila University of Medicine and Pharmacy, Bucharest, Romania; 4 Flavocure Biotech Inc, Baltimore, Maryland USA; 5 Department of Biomedical Engineering, Indian Institute of Technology Hyderabad, Hyderabad, India

## Abstract

Clinical cancer treatment modalities include radiation as one of the first-line therapies used for treating almost two-thirds of cancer patients. Combinational therapy for cancer is becoming extremely popular, with multiple therapies and their pharmacological effects expected to provide a synergistic outcome. The nanotechnology-based combinational therapeutic approach is emerging as a more effective strategy, for its advantages include simultaneous loading of multiple drugs, on-demand drug delivery controlled by external or internal stimulus, targeting a particular site, and the potential to combine physical treatment modalities (like radiation, thermal therapies, etc.) with chemical interventions (like chemotherapy, immunotherapy, etc.). We report a combination of radiotherapy and chemotherapy mediated by a multifunctional lipo-polymeric hybrid nanosystem coated with gold, demonstrating the three different functionalities using a single nanosystem: a) radio sensitization, b) radiation-triggered delivery of drugs, and c) application as an X-ray/CT contrast agent. The lipo-polymeric hybrid nanoparticles, synthesized using a modified hydrogel isolation method, were loaded with a natural plant-derived anti-cancer agent “Caflanone.” These nanoparticles were further subjected to *in-situ* reduction for a surface coating of gold, which provided enhanced radiosensitivity, radiation triggered drug delivery and X-ray/CT imaging. This approach using a multifunctional nanosystem leverages the biocompatibility of the lipo-polymeric hybrid system for the loading of drugs, precise spatiotemporal controllability of radiation for drug release, and the cytotoxicity of the plant-derived anti-cancer agent “Caflanone.” A significant therapeutic efficacy in vitro against breast cancer (*p* = 0.0002), pancreatic cancer (*p* < 0.0001), and glioblastoma (*p* < 0.0001) was demonstrated with the combinational approach. The application of the nanosystem as an X-ray/CT contrast agent has been shown in vivo in tumor-bearing mice and the safety profile and histopathology evaluated in healthy mice showed no adverse effects. A significant increase (*p* = 0.01) in the survival of breast tumor-bearing mice treated with a combinational approach was also demonstrated. The engineered multifunctional nanoparticles enhanced the radiation therapy and triggered the drug release at the tumor site, triggering the action of encapsulated chemotherapeutic agents while providing image guidance.

**Supplementary Information:**

The online version contains supplementary material available at 10.1186/s12943-025-02266-1.

## Introduction

X-ray radiotherapy remains a standard treatment approach for numerous cancers [1–4]. However, standard radiotherapy is limited by damage to healthy tissues and the development of radiation resistance. Imaging plays an essential role in the planning and delivery of radiotherapy. The recent advances in imaging led to the development of advanced and adaptative radiotherapy techniques allowing the design of personalized treatments and implementation of highly conformal treatment for delivering adequate doses confirming to the target and sparing the surrounding normal tissues [5–7]. Radiosensitizers, chemical or biological compounds that absorb and make cancer cells more susceptible to radiation, are known to improve therapeutic efficacy and reduce the side effects of radiotherapy [8–10]. The rapid development of nanotechnology offers potential therapeutic strategies employing nanoparticles with varying sizes, morphologies and functionalities that can play a crucial role in enhancing radiation therapy by acting as the carriers of radiosensitizers or radiosensitizers themselves [10–13]. Nanosystems offer the advantages of targeted drug delivery, prolonged plasma circulation [14], improved biodistribution of drugs and bioavailability, and reduced adverse effects [15].

High Z metal nanoparticles are known to enhance the therapeutic ratio of radiation therapy by augmenting effective doses within tissues through increased secondary electrons (Photo, Compton, and Auger electrons) and free radical production in the tumor microenvironment [4, 9, 11, 16–19]. Gold nanoparticles are considered ideal radiosensitizers owing to their high density, large X-ray absorption coefficient, unique physicochemical properties [20–22], size-dependent optical and electronic characteristics [23–25], biocompatibility, easier synthesis, and surface functionalization approaches [19, 26, 27]. Following the pioneering studies by Hainfield et al. using gold nanoparticles in combination with X-rays, demonstrated eradication of EMT-6 mammary xenograft tumors with a survival rate of 86%, [19, 28, 29] gold nanoparticles have been studied extensively for imaging, and therapy in combination with other chemotherapeutic agents for potential synergy in activity [7, 30, 31].

Combining radiotherapy and chemotherapy has received immense attention for improved therapeutic efficacy [15, 32]. Chemotherapy drugs create a systemic effect, inhibiting tumor growth and destroying cancer cells [10], but are also limited by bioavailability, lack of specificity, and limited circulation time [33]. The limiting factors of both chemotherapy and radiation therapy motivate multimodal therapy using a combination of both [11, 34]. Combinational therapy is a promising approach for enhanced therapeutic outcomes by targeting multiple pathways and improving treatment outcomes, decreasing dosages, reducing adverse effects, and decreasing drug resistance [35, 36]. A strategic approach will include a combination of radiotherapy and chemotherapy using multifunctional nanoparticles designed to enhance radio sensitization and deliver drugs while mitigating side effects on normal tissue.

Multifunctional nanoparticle-based systems are emerging as a robust approach for co-loading multiple active agents, improving bioavailability and drug solubility, providing prolonged half-life, elevated tissue penetration, and reduced adverse effects [35, 37, 38]. Radiation-responsive multifunctional nanosystems can be designed to transport drugs to tumor tissues and enable on-demand triggered drug release at the tumor sites [32]. The X-rays are known for their deep tissue penetration and high conformal delivery [33], which can be applied as an external stimulus for achieving controlled drug release in deep-seated tumors. These radiation-sensitive nanoparticles would provide spatial and temporal controllability [34] and can release the drugs from the nanoparticles injected intratumorally, followed by treatment with X-rays exclusively at the tumor site, reducing the adverse effects.

Liposomes [39] and polymers [40] have been reported to deliver drugs using radiation as an external trigger. However, the multifunctionality of nanoparticles can be further harnessed to extend the combination of radiotherapy and chemotherapy and facilitate imaging, thereby overcoming the restrictions or limitations of individual treatment modalities and the requirement of multiple therapeutic or imaging agents. In this context, we report a lipo-polymeric hybrid nanosystem coated with gold, demonstrating its multifunctionality as 1) a radiosensitizer, 2) a radiation-responsive nanosystem for drug delivery, and 3) an X-ray/CT contrast agent. We report the synthesis of the nanosystem and optimize the surface coating with gold to achieve maximum radiosensitivity; load the nanosystem with a natural anti-cancer agent, Caflanone (provided by Flavocure Biotech Inc, USA), and understand its radiation-triggered release and disintegration of nanoparticles. We further investigated and demonstrated the in vitro therapeutic efficacy using multifunctional nanoparticles in three cancer cell types: breast, pancreatic, and brain. We further demonstrated the safety profile of these nanoparticles and their application for X-ray/CT imaging contrast in vivo. We also showed a significant therapeutic efficacy: reduced tumor volume and increased survival in vivo with the nanoparticle-mediated combinational treatment approach.

## Materials and methods

### Materials

The lipid 1,2-dioleoyl-sn-glycero-3-phospho-L-serine (sodium salt) (DOPS-Na) was purchased from Avanti Polar Lipids, U.S.A. Polyethylene glycol (PEG), polyvinylpyrrolidone (PVP), Sodium chloride, Calcium chloride, Crystal violet, Ascorbic acid, Hydrogen Tetrachloroaurate(III)/Chloroauric acid (HAuCl_4_.3H_2_O), Fluorescein Diacetate (FDA), 2,7-dichlorodihydrofluorescein diacetate (DCFHDA), Propidium Iodide (PI), MTT (3-(4,5-Dimethylthiazol-2-yl)−2,5-Diphenyltetrazolium Bromide) were purchased from Millipore Sigma, USA. Phosphate buffer pH 7.0, Accutase, Dulbecco's Modified Eagle Medium (DMEM), Roswell Park Memorial Institute Medium (RPMI), and Calf Serum (US origin) were purchased from ATCC, USA. Caflanone was provided by Falvocure Biotech Inc, USA.

### Characterization

The absorbance and fluorescence were read by the microplate reader (Spectramax, Molecular Devices, LLC, USA). The size and zeta potential were measured by a particle size analyzer (Malvern Nano ZS, Malvern Panalytical Ltd, UK). The morphology of the nanoparticles and the elemental analysis was recorded using Scanning electron microscopy (JEOL IT700HR, JEOL, USA) and Transmission Electron Microscopy (Hitachi 7600 TEM, Hitachi Hi-Technologies Corporation, Japan). The cell imaging was performed using a fluorescence microscope (EVOS M7000, Thermofisher Scientific, USA). The clonogenic assay plates were imaged using an automatic colony counter (Scan 4000, Interscience, France). The cells and the nanoparticles were irradiated using an X-RAY irradiator (MultiRad 225, Precision X-ray Irradiation, U.S.A, 225kVp and 17 mA) and (CIXD, Xstrahl Inc, USA, 220kVp and 13 mA). The mice were irradiated (220kVp, 13 mA) using the Small Animal Radiation Research Platform (SARRP, Xstrahl Inc, USA). X-ray/CT imaging was performed using Nanoscan PET/CT (Mediso, USA).

### Synthesis of nanoparticles

- Lipo-polymeric hybrid nanoparticles (PDPC NPs):

The nanoparticles were synthesized as follows: liposomes of DOPS-Na with or without the drug Caflanone were prepared using a thin film hydration technique. The lipo-polymeric hybrid nanoparticles were prepared using a modified hydrogel isolation technique [41, 42]. Briefly, 1.98 g of PEG and 0.52 g of PVP were made into two solutions with Milli-Q water. Liposomes (10 mg/mL) were added to the PEG solution under stirring, which was injected into the PVP solution under constant stirring. Calcium chloride solution (100 mM) was added dropwise, and the mixture was allowed to stir at room temperature for one hour. The nanoparticles were washed twice with buffer solution (1 mM CaCl_2_, 150 mM NaCl) and recovered by centrifugation. The nanoparticles were dispersed in buffer and stored at 4 °C.

b) Gold-coated lipo-polymeric hybrid nanoparticles (PAu NPs):

The lipo-polymeric hybrid nanoparticles were coated with gold by a chemical reduction of HAuCl_4_.3H_2_0 with Ascorbic acid [41, 42]. Briefly, 2 mg/ml PDPC NPs were mixed with HAuCl_4_.3H_2_0, followed by the addition of ascorbic acid (10 mM). The solution first turned colorless and slowly to dark brown, indicating the formation of gold-coated lipo-polymeric hybrid nanoparticles (PAu NPs). For the synthesis of PAu_5_, PAu_10_, PAu_15_, and PAu_20_ NPs, the same procedure was followed with the concentrations of HAuCl_4_.3H_2_0, depending on the type of nanoparticle i.e., 5 mM, 10 mM, 15 mM, and 20 mM of HAuCl_4_.3H_2_0 were used for PAu_5_, PAu_10_, PAu_15_, and PAu_20_ NPs, respectively.

c) Loading of Caflanone into lipo-polymeric hybrid nanoparticles (PC NPs) and their surface coating with gold (PCAu NPs):

Firstly, liposomes loaded with Caflanone were prepared by thin-film hydration technique [43]. Briefly, 2 mg of Caflanone was dissolved in methanol and subjected to bath sonication for dispersion of the drug. The lipid (10 mg) was dissolved in chloroform, and the drug solution was added to the lipid solution. The solvents were evaporated using a rotary evaporator, and MilliQ water (18Ω) was added for hydration. Following the thin film hydration, the liposomal solution with the drug was collected (10 mg/ml) and subjected to probe sonication for 15 min. The Caflanone-loaded lipo-polymeric hybrid nanoparticles (PC NPs) were synthesized using the modified hydrogel isolation technique. The PC NPs were sonicated for about 15 min using a probe sonicator. For surface coating with gold, 2 mg/ml PC NPs were mixed with HAuCl_4_.3H_2_0 (15 mM), followed by ascorbic acid (10 mM). The solution initially turned colourless and then turned to dark brown color within a few minutes.

### Encapsulation efficiency of the nanoparticles

The PC NPs were evaluated for their encapsulation efficiency using the peak absorbance maxima of the drug [44]. A calibration curve was plotted with concentrations of Caflanone ranging from 0–150 µg. For the calculation of encapsulation efficiency, 200 µL of PC NPs were centrifuged, the pellet was dispersed in 1 mL of DMSO, and the absorbance at 350 nm corresponding to Caflanone was recorded. The amount of Caflanone loaded was calculated using the regression equation y = 0.0206x + 0.0835 (R^2^ = 0994) and the encapsulation efficiency was calculated [38].

### Radiation triggered release of caflanone from nanoparticles

The radiation triggered release [39] of Caflanone from the nanosystem was studied using four batches of PC NPs, and PCAu NPs. The PC NPs served as a control. 100 µL of PC NPs and PCAu NPs were subjected to various doses of radiation, i.e., 0, 10, 20, and 30 Gy. Following the radiation, the absorbance at 350 nm (peak absorbance maxima of Caflanone) was recorded. The same set of samples, PCAu NPs, were also used for analysis using Transmission Electron Microscopy (TEM), to understand the disintegration of the nanoparticles with radiation [34, 41, 45].

### In vitro studies

#### Cell culture

Murine breast carcinoma (4T1) and glioblastoma cells (GL261) were obtained from the ATCC, USA. The pancreatic cancer cell line (KPC) was obtained from Cancer Research Technology Limited, UK. The cell lines were cultured in DMEM/RPMI medium supplemented with 10% (v/v) Fetal bovine serum (FBS) and penicillin/streptomycin. The cells were cultured in a humidified atmosphere containing 5% CO_2_, with temperature maintained at 37 °C under sterile conditions.

The in vitro studies were performed in three different cancer cell lines of mice origin: breast cancer (4T1), pancreatic cancer (KPC), and glioblastoma (GL261). The therapeutic efficacy was evaluated using the colony forming assay/clonogenic assay (CFU assay) and MTT assay. The intracellular ROS was evaluated using the DCHFDA assay. A live/dead assay was also performed to visualize the live and dead cells using FDA/PI staining.

- Colony forming assay (CFU assay): Briefly, 200 cells per plate/well were seeded the day before treatment with nanoparticles. The drug Caflanone, gold-coated lipo-polymeric hybrid nanoparticles (PAu NPs), and gold-coated Caflanone loaded lipo-polymeric hybrid nanoparticles (PCAu NPs) were dispersed in cell culture media (with the concentration of Caflanone: 1.25 µg) and were added to the cells. Following the incubation with drug/nanoparticle suspensions for 24 h, the cells were subjected to various doses of radiation: 2 Gy, 4 Gy, 6 Gy, 8 Gy, and 10 Gy. After ~ 10 days, each plate/well was washed twice with PBS, fixed with ice-cold methanol, and stained with 0.5% crystal violet [46]. The stained cell colonies were hand-counted, with a colony defined as a distinct group of cells with 50 or more cells. The images of the plates were captured using an automatic colony counter.
- MTT assay: Briefly, 2 × 10^4^ cells were seeded in a 96-well plate, and the cells were treated with nanoparticles diluted in cell culture media. Following incubation with the nanoparticles for 24 h, the cells were subjected to radiation (10 Gy). The MTT assay was performed the next day. The MTT reagent (3-(4,5-dimethylthiazol-2-yl)−2,5-diphenyl-2H-tetrazolium bromide), dissolved in serum-free media (5 mg/mL) was added to the cells (5 µg/ well) and left undisturbed for three hours. DMSO (100µL) was added to each well, and the plate was read for absorbance at 570 nm with reference at 650 nm. The viability was calculated as compared to controls (untreated cells) [43, 47, 48].
- DCFHDA assay: The intracellular ROS was evaluated using this qualitative and quantitative assay [43]. 2 × 10^4^ cells were seeded in a 96-well plate. The cells were treated with nanoparticles diluted in cell culture media after 24 h. Following incubation with the nanoparticles for 24 h, the cells were subjected to radiation (10 Gy) and were incubated for another 24 h. The DCFHDA (25 μM) solution in serum-free media was added to the wells and incubated for about 45 min. The fluorescence intensity was measured at 535 nm (for an excitation at 485 nm) using a microplate reader. The cells were imaged using a fluorescence microscope. Untreated cells were considered as controls.
- Live/Dead assay: FDA/PI staining was used to assess therapeutic efficacy qualitatively. Briefly, 1 × 10^5^cells were plated in a 6-well plate. The nanoparticles were added to the cells and incubated for 24 h. The cells were subjected to radiation (10 Gy) and incubated for another 24 h. The cells were incubated with FDA and PI for about 5 min. The cells were washed with PBS and imaged using a fluorescent microscope to observe the live cells and dead cells fluorescing in green and red, respectively [49].

### In vivo studies

#### Tumor models

The in vivo studies followed the guidelines revised and permitted by the Johns Hopkins University Animal Care and Use Committee (ACUC) for protocol #MO21M281, approved on 6 October 2021. The experiments were conducted in female Balb/C mice and female C57BL/6 mice (4–6 weeks) procured from Jackson Laboratories, U.S.A.

The in vivo studies were performed in two mice models: the breast cancer model (developed using 4T1 cells) [41] and the pancreatic cancer model (developed using KPC cells) [50]. The breast cancer model was developed by injecting 2 × 10^5^ 4T1 cells/animal subcutaneously to the dorsal flank region of female Balb/C mice. The pancreatic mice model was developed by injecting 1.5 × 10^5^ KPC cells/animal subcutaneously to the dorsal flank region of female C57BL/6 mice. When the tumors reached an appropriate size, the animals were randomly grouped for imaging and therapy.

- X-ray/CT Imaging and analysis

This experiment was performed on mice with a single subcutaneous pancreatic tumor on the dorsal flank. The PCAu NPs (with conc. of Caflanone: 700 μg; gold: 2.95 mg) were intratumorally injected, followed by focal radiation of 12 Gy, targeted to the tumor site. Five mice were imaged before and after injecting the nanoparticles and after receiving radiation up to Day 35. The CT volumes of mice were acquired at multiple time points using consistent acquisition parameters. These volumes were then processed in MITK to annotate various anatomical structures, including the entire mouse body within the scanning cage, bones, tumor volumes, and nanoparticles. Volumetric visualizations were subsequently generated from these annotations. The nanoparticle contrast over the treatment period was estimated by contouring the CT images using the semi-automated contouring software Medical Imaging Interaction Toolkit (MITK) version 2022.04. Full-body CT volumes of the mice were acquired with the Nanoscan PET/CT (Mediso, USA) system, providing a resolution of [0.16 mm, 0.16 mm, 0.16 mm]. The analysis was conducted using Python 3.9 and SimpleITK 2.3. Contouring of the mice's bodies, bones, tumors, and nanoparticles was performed in MITK, while 3D model generation and visualization were accomplished using Slicer version 5.2.1 [51, 52].

b. Safety profile of the nanoparticles

The safety profile of the nanoparticles was evaluated in healthy female Balb/C mice. Two groups of mice, control, and treatment groups were used for this experiment. To evaluate the safety profile, the body weight, plasma, serum, complete blood panel, and histopathology of major organs (Kidneys, Lungs, Liver, Spleen) were monitored [34, 53] with the treatment of PCAu NPs (concentration of Caflanone: 700 µg, gold: 2.95 mg) for 3 h, 7 days, 14 days and 30 days and compared with the control mice that received no treatment.

c. Therapeutic efficacy

This study was performed in two different sets of mice. The first experiment was performed in mice with a single subcutaneous breast tumor on the dorsal flank. The mice were divided into the control group (that received no treatment, *n* = 4) and the treatment group (that received PCAu NPs and radiation 12 Gy, *n* = 4). The PCAu NPs (with conc. of Caflanone: 700 μg; gold: 2.95 mg) were intratumorally injected, followed by radiation of 12 Gy. The mice were imaged before receiving radiation and after the treatment up to Day 28. The tumor volume and survival of the mice were monitored.

The second experiment was performed in mice with two subcutaneous tumors, one each on the dorsal flank, representing a metastatic tumor model [54]. The mice were randomly grouped into two groups: a control (no treatment, *n* = 3) and a treatment group (that received PCAu NPs and radiation 12 Gy, *n* = 4). Only one of the tumors in the treatment group was injected with PCAu NPs and subjected to radiation (12 Gy). The secondary tumor was not treated but monitored for its volume throughout the study. The tumor volumes and survival were monitored, and the graphs were plotted.

### Statistical analysis

The statistical analysis was performed wherever necessary. GraphPad prism was used for the analysis. Student t-tests and two-way ANOVA followed by Tukey’s test were used to understand the significance. For the survival of the mice, the Log-rank test was used. The *p* ≤ 0.0001: ****, *p* ≤ 0.001: ***, *p* ≤ 0.01: **, *p* ≤ 0.05: *

## Results and discussions

**Fig. 1:**
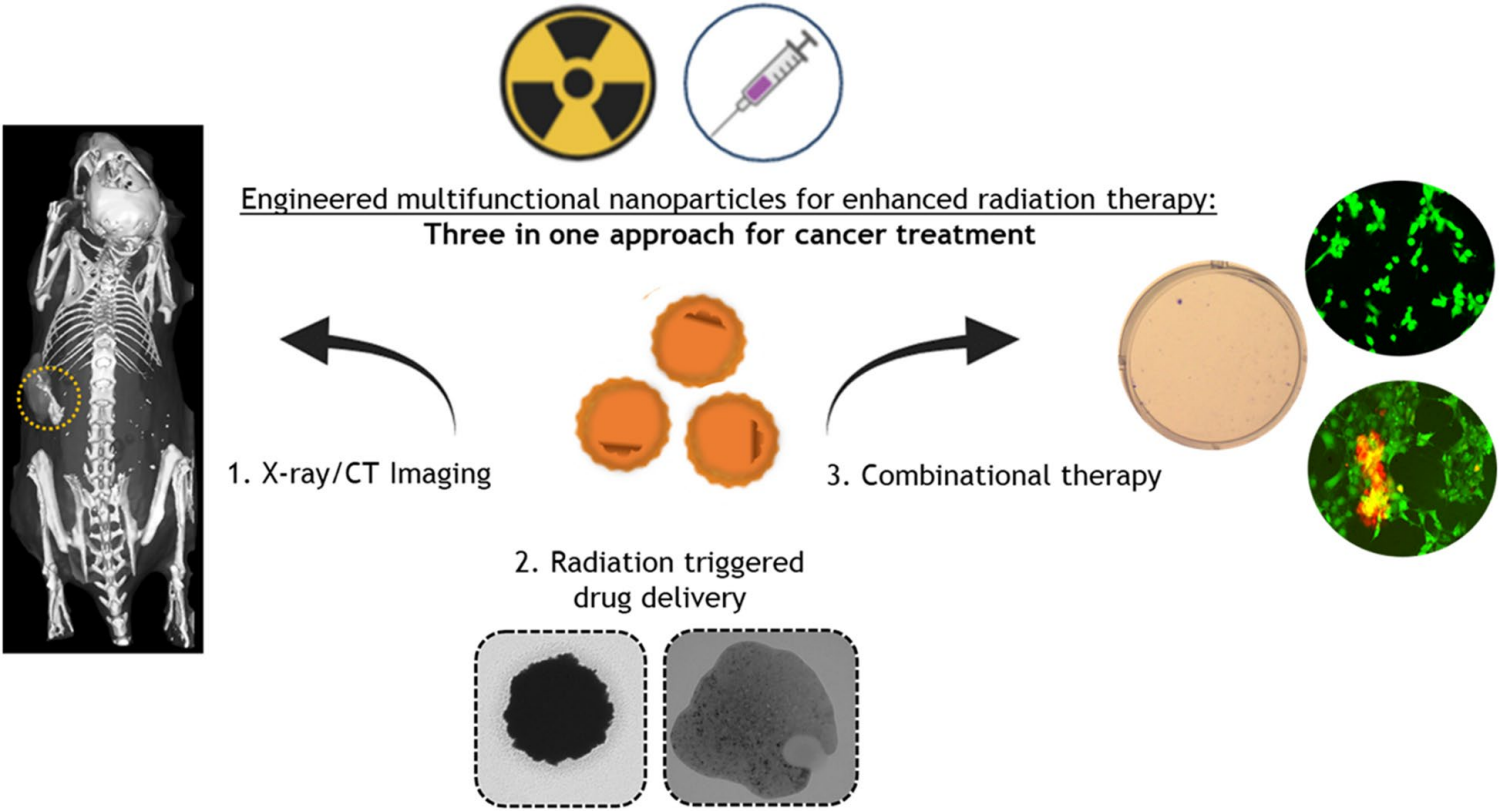
Schematic showing the application of multifunctional nanoparticles for imaging and combinational therapy

Lipo-polymeric hybrid nanosystems are known for their dual-structured character and offer combined advantages of lipids and polymers while overcoming each other’s limitations [55]. In this study, we demonstrate the multifunctional capabilities of a lipo-polymeric hybrid through a series of in vitro and in vivo studies. Figure 1 shows the schematic illustrating the multifunctional applications of lipo-polymeric hybrid nanosystems.

### Synthesis and characterization

**Fig. 2:**
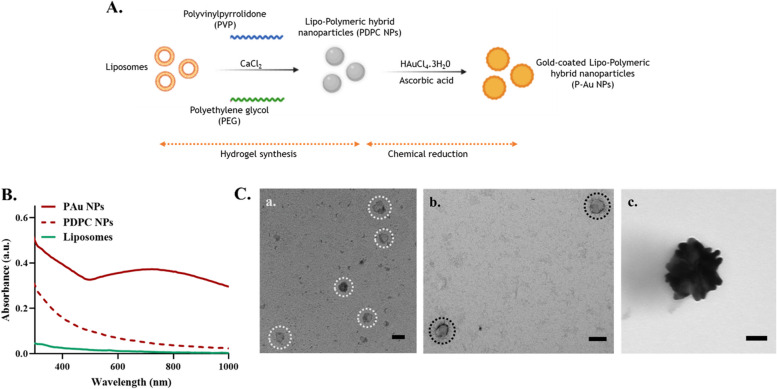
Synthesis and characterization. **A** Schematic showing the synthesis of gold-coated lipo-polymeric hybrid nanoparticles (NPs). **B** Absorbance spectra of the nanoparticles, **C** TEM imaging of a. liposomes, b. Lipo-polymeric hybrid nanoparticles (PDPC NPs) and c. gold-coated lipo-polymeric hybrid nanoparticles (PAu NPs). (*Scale bar corresponds to 100 nm)

The lipo-polymeric nanoparticles (PDPC NPs) were synthesized by a modified hydrogel-isolation technique. The lipo-polymeric hybrid nanoparticles are further coated with gold by a chemical reduction using ascorbic acid, forming PAu NPs (Fig. 2A). The absorption spectra (Fig. 2B) of liposomes and lipo-polymeric hybrid nanoparticles showed no specific absorbance. In contrast, the gold-coated nanoparticles (PAu NPs) show a broad absorption peak in the near-infrared region (600–900nm), confirming the surface coating with gold [41]. The mean hydrodynamic diameter of liposomes and lipo-polymeric hybrid particles as measured by dynamic light scattering were 76.49 ± 10.53 nm, and 96.21 ± 6.280 nm, respectively. The size of the lipo-polymeric hybrid nanoparticles coated with gold was recorded as 136.2 ± 2.53 nm. The TEM images (Fig. 2C) show the size and morphology of the nanoparticles, with the average sizes correlating with the sizes measured by dynamic light scattering. The liposomes and lipo-polymeric hybrid nanoparticles were uniform and spherical, and the surface coating with gold can be evidenced from Fig. 2Cc. The SEM image (Figure S1A) shows the homogenous size and shape of PAu NPs. The energy-dispersive X-ray spectroscopic analysis of PAu NPs (Figure S1B) showing the presence of gold (Au), further confirmed the surface coating with gold.

### Radiosensitization of cancer cells

**Fig. 3:**
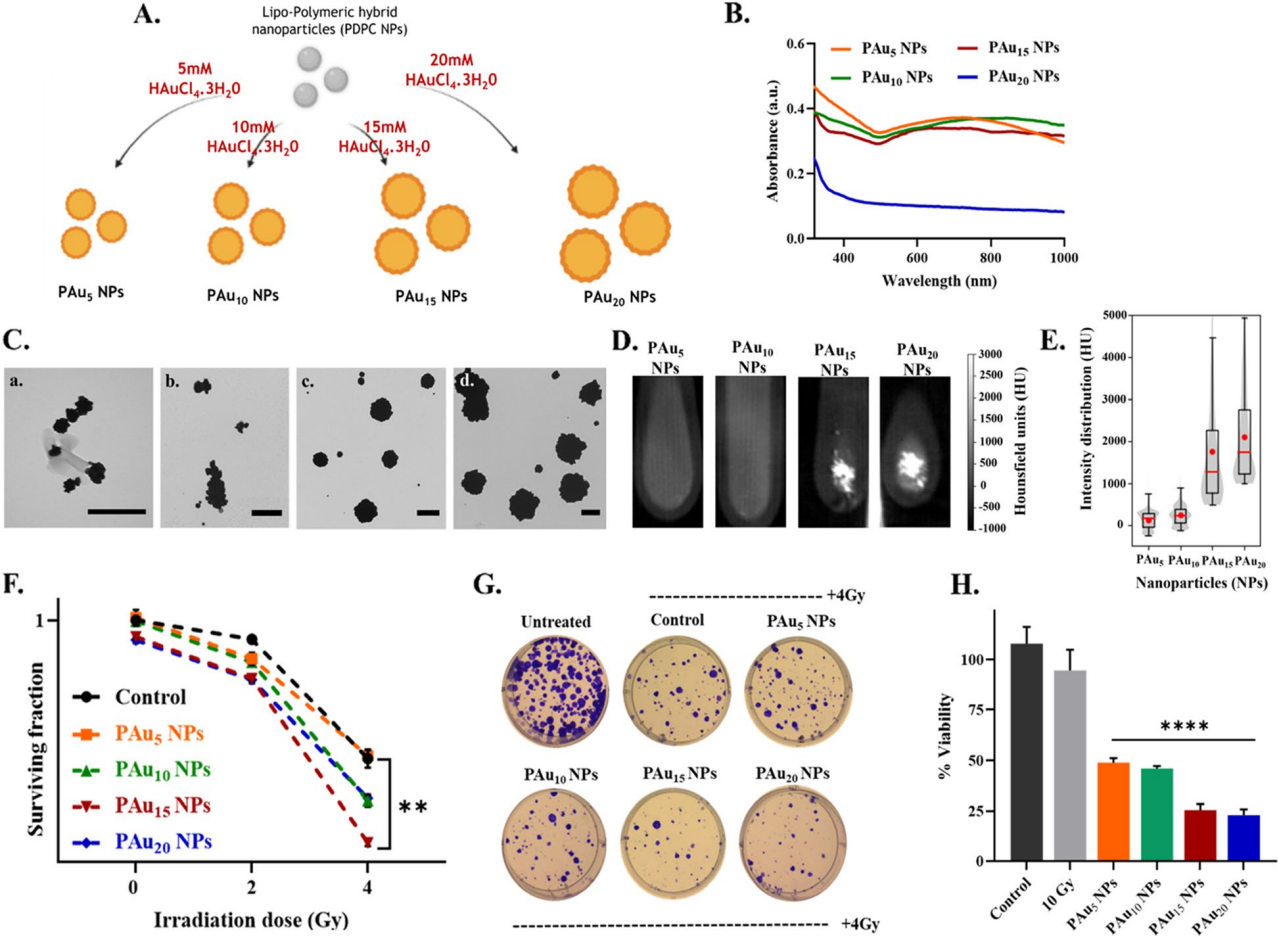
Lipo-polymeric nanoparticles with varying concentrations of gold. **A** Schematic showing the synthesis of nanoparticles (NPs). **B** Absorbance spectra, **C** TEM imaging of nanoparticles a. PAu_5_, b. PAu_10_, c. PAu_15_, d. PAu_20_ (*Scale bar corresponds to 500 nm), **D** Image showing the X-ray/CT contrast of the nanoparticles, **E** Quantified values of the X-ray/CT contrast of the nanoparticles, **F** Survival fraction of the 4T1 cells (breast cancer), **G** Images showing the clonogenic assay (CFU), **H** MTT assay in 4T1 cells with nanoparticles and radiation (10 Gy)

The radiation sensitivity provided by high-Z metals is very well-established [56, 57]. We evaluated if the surface-coating of PDPC NPs with gold could improve the sensitivity of the cancer cells to radiation and provide X-ray/CT contrast. The PDPC NPs have been coated with various concentrations (5 mM, 10 mM, 15 mM, 20 mM) of Hydrogen Tetrachloroaurate(III)/Chloroauric acid HAuCl_4_.3H_2_0 for the preparation of gold-coated lipo-polymeric hybrid nanoparticles: PAu_5,_ PAu_10_, PAu_15_, and PAu_20_ NPs as shown in the illustration Fig. 3A. The absorbance spectra (Fig. 3B) confirmed the surface coating with gold. The mean hydrodynamic diameters of PAu_5,_ PAu_10_, PAu_15_, and PAu_20_ NPs were 136.2 ± 2.53 nm, 248.4 ± 10.93 nm, 308.7 ± 10.42 nm, and 455 ± 6.451 nm, respectively. The TEM images of the nanoparticles (Fig. 3C) show the increased size (Figure S1C) and uniformity of the nanoparticles with increasing concentrations of gold precursor.

The nanoparticles were investigated for their X-ray/CT contrast (Fig. 3D & S1D). The intensity of the X-ray/CT contrast of the nanoparticles was noted to increase remarkably (Fig. 3E), with increased concentrations of Au [58]. These nanoparticles were further investigated for their radiosensitization in breast cancer cells (4T1). The clonogenic survival with nanoparticles and radiation (4 Gy) (Fig. 3F&G and Figure S2) showed significant (*p* = 0.084) cytotoxicity with PAu_15_ NPs as compared to control. The number of colonies with the treatment of PAu_15_ NPs and 4 Gy were significantly lower. The viability of the cells was also evaluated using MTT assay and all the nanoformulations showed a significant decline (*p* < 0.0001) in the cell viability, demonstrating the effect of radiosensitization. Amongst all the formulations, PAu_15_ NPs showed better cytotoxicity as compared to the PAu_5_ and PAu_10_ NPs, for uniform doses of radiation, correlating with the results of the CFU assay. These results validate the application of the gold-coated lipo-polymeric hybrid nanoparticles as radiosensitizers and X-ray/CT imaging agents. The PAu_15_ NPs are chosen for all further studies, for their enhanced radiosensitivity and better X-ray/CT contrast and would be referred to as PAu NPs or blank nanoparticles.

### Radiation triggered drug delivery

**Fig. 4:**
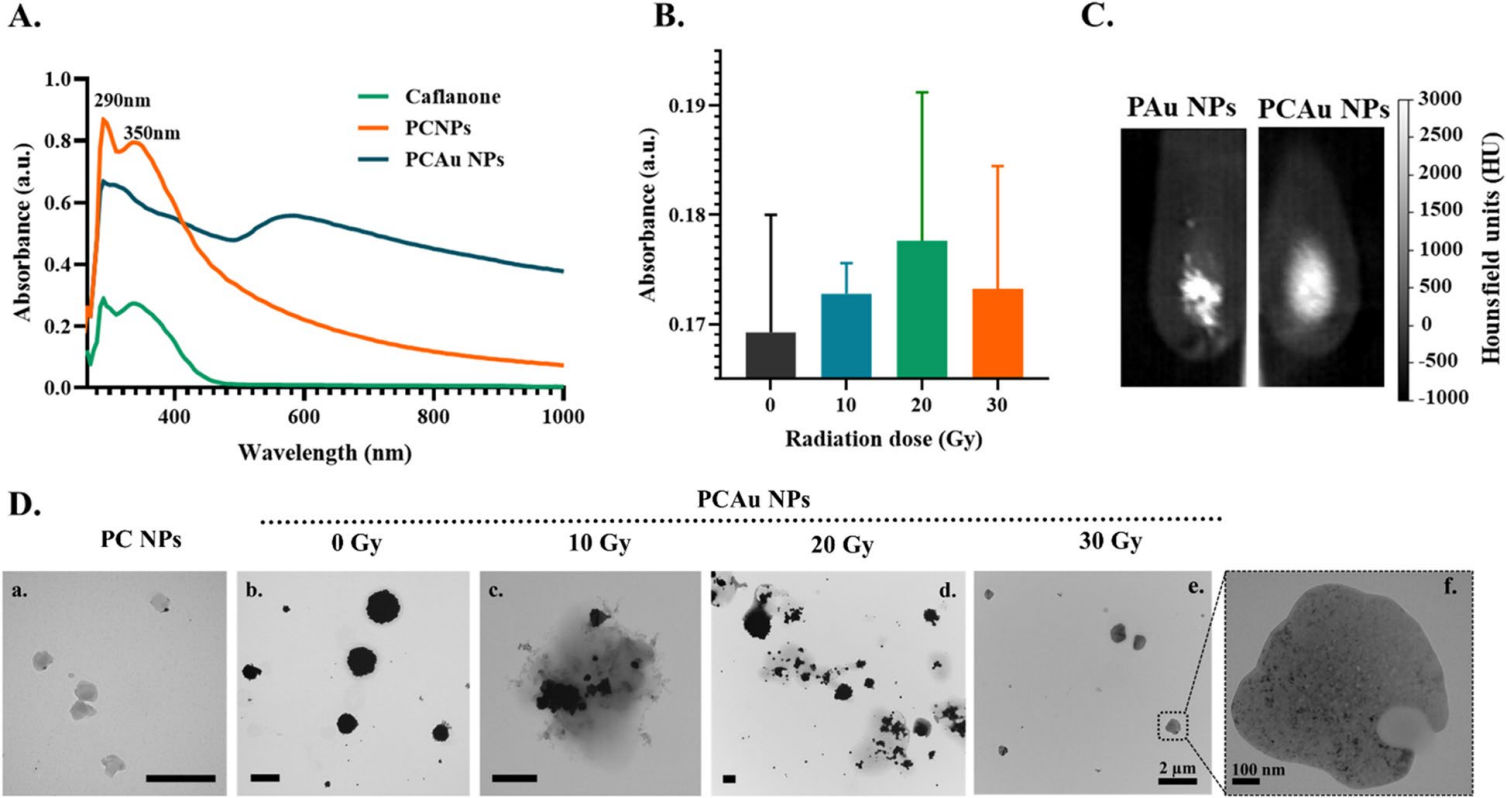
Drug (Caflanone) loading and release with radiation (X-rays) **A** Absorbance spectra of the drug (Caflanone) and drug-loaded nanoparticles. **B** Drug release from PCAu NPs with radiation, **C** Image showing the X-ray/CT contrast of the blank nanoparticles (PAu NPs) and nanoparticles loaded with drug (PCAu NPs), **D** TEM images showing the a). PC NPs, b). PCAu NPs, (c-e), PCAu NPs subjected to 10, 20, and 30 Gy doses of radiation respectively, and f. High magnification image showing complete disintegration of the nanoparticle with radiation (*Scale bar for TEM images in D(a-d) corresponds to 500 nm)

Caflanone is a flavonoid derivative of *Cannabis Sativa L*. and has been reported for its therapeutic efficacy in treating pancreatic cancer in pre-clinical models [59]. The effect of Caflanone has been evaluated in both breast and pancreatic cancer cells (Figure S3 A&B). The lipo-polymeric hybrid nanoparticles (PDPC NPs) were loaded with Caflanone, forming PC NPs. The encapsulation efficiency of Caflanone within PDPC NPs was estimated to be about 64.43 ± 1.9% (Figure S3C). These nanoparticles were further coated with gold, forming PCAu NPs with an average size of 271.3 ± 13.67 nm and a surface charge of −19.77 ± 4.63. Consistent with successful encapsulation, the absorption spectra in Fig. 4A showed the peaks corresponding to Caflanone (290 nm and 350 nm) in PC NPs, and the broad absorption peak in the NIR (Near infrared) region of PCAu NPs confirms the surface coating with gold. The SEM image (Figure S4A) shows the uniform PCAu NPs, and their elemental analysis (Figure S4B) further confirms the presence of gold. The size and shape of the PC NPs could be visualized from the TEM image, as shown in Fig. 4Da.

Following the successful encapsulation of Caflanone, we next investigated the X-rays/radiation-triggered release of the Caflanone. The PC NPs were considered controls: as we hypothesized, the X-rays interacted with gold coating on the surface, disintegrating the nanoparticles and facilitating their release, and hence nanoparticles without any gold coating were considered controls. With increasing doses of radiation, an increase in the absorption of the Caflanone (Fig. 4B) was recorded for PCAu NPs, indicating the drug release [39]. For the same doses of radiation, no notable change in the absorption was recorded for PC NPs (Figure S5A), indicating that the X-rays interacted with the gold coating, facilitating the drug release. The same has been observed using Transmission electron microscopy images, as shown in Fig. 4D (b-f). The PC NPs were almost spherical (Fig. 4Da), and PCAu NPs (Fig. 4Db) showed a dark contrast, confirming the surface coating with gold. Figure 4D (c-f) shows the changes in the morphology of the nanoparticles with varying doses of radiation (10, 20, and 30 Gy), respectively. The disintegration of nanoparticles increased with the radiation dose, and for the highest dose of radiation, i.e., 30 Gy, the nanoparticles disintegrated into smaller gold nanoparticles (dark spots). This disintegration with radiation hints at the possibility that these nanoparticles might as well be cleared from the system following the therapy [45]. The PCAu NPs were further investigated for their X-ray/CT contrast and compared to PAuNPs, i.e., gold-coated lipo-polymeric hybrid nanoparticles without any drug. Figure 4C (and Figure S5 B&C) shows the X-ray/CT contrast of PCAu NPs, comparable to PAu NPs, indicating the drug loading has not compromised the nanoparticle’s ability to function as an X-ray/CT contrast agent.

### In vitro studies: the therapeutic effect of combinational approach

The PCAu NPs were further investigated for their combinational therapeutic outcome against three different cancer cell lines: breast cancer (4T1), pancreatic cancer (KPC), and glioblastoma (GL261), owing to their enhanced radiosensitivity and radiation-responsive drug release. We performed clonogenic (colony-forming/CFU) assay, MTT assay and DCFHDA assays to evaluate the effect of combined radiation therapy and Caflanone compared to individual treatments.

**Fig. 5:**
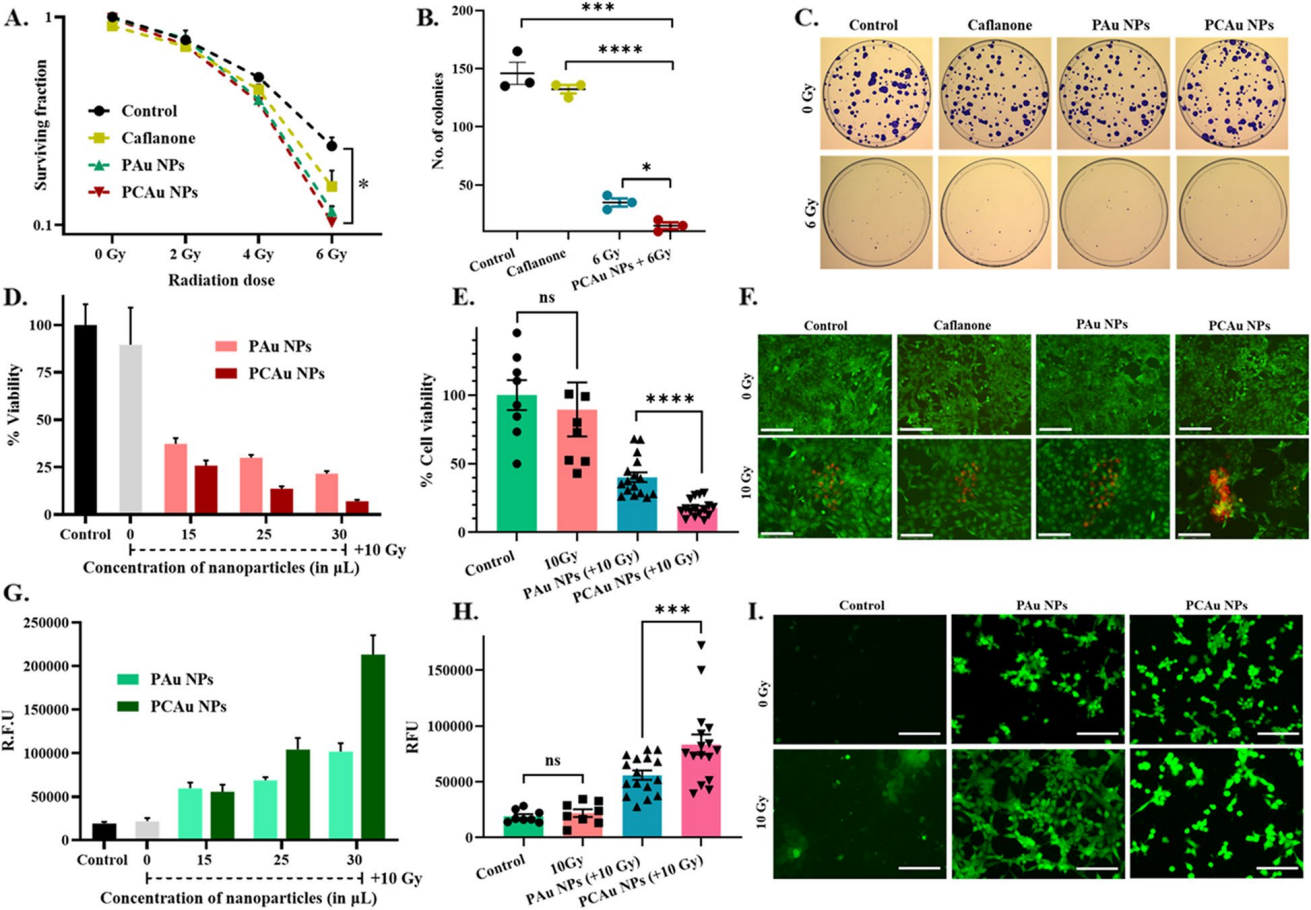
In vitro therapeutic efficacy against breast cancer cells (4T1) **A** Survival fraction of the 4T1 cells (breast cancer), **B** Comparison of combinational therapy and individual modalities, **C** Images showing the clonogenic assay (CFU assay) with nanoparticles and radiation (6 Gy), **D** MTT assay in 4T1 cells with nanoparticles and radiation (10 Gy), **E** MTT assay showing the significant difference in viability with PAu NPs and PCAu NPs in combination with radiation (10 Gy), **F** Live/Dead assay showing the effect of treatment with nanoparticles and radiation (FDA stains live cells in green and PI stains dead cells in red) (*Scale bar corresponds to 150 µm), **G** DCFHDA assay in 4T1 cells, **H** DCFHDA assay showing the significant difference between blank (PAu NPs) and drug-loaded nanoparticles (PCAu NPs) in combination with radiation (10 Gy), **I** Microscopic images showing the intracellular ROS (*Scale bar corresponds to 150 µm). Statistics: student t-test; *p* ≤ 0.0001: ****, *p* ≤ 0.001: ***, *p* ≤ 0.01: **, *p* ≤ 0.05: *

- Breast cancer (4T1):

The clonogenic survival assay (CFU assay) showed a significant difference in the survival fraction, as compared to only radiation (*p* = 0.0114) (Fig. 5A and Figure S6). The combination (PCAu NPs & radiation) yielded a > tenfold reduction in the number of colonies formed as compared to control (Fig. 5B). The PCAu NPs showed significant therapeutic outcomes compared to only the drug-treated group (Caflanone; *p* = 0.0003) and the only radiation-treated group (6 Gy; *p* = 0.0114), demonstrating the enhanced effect of the nanoparticles. Figure 5C (and Figure S6) show the different treatment groups with and without radiation and their effect on the formation of colonies.

**Fig. 6:**
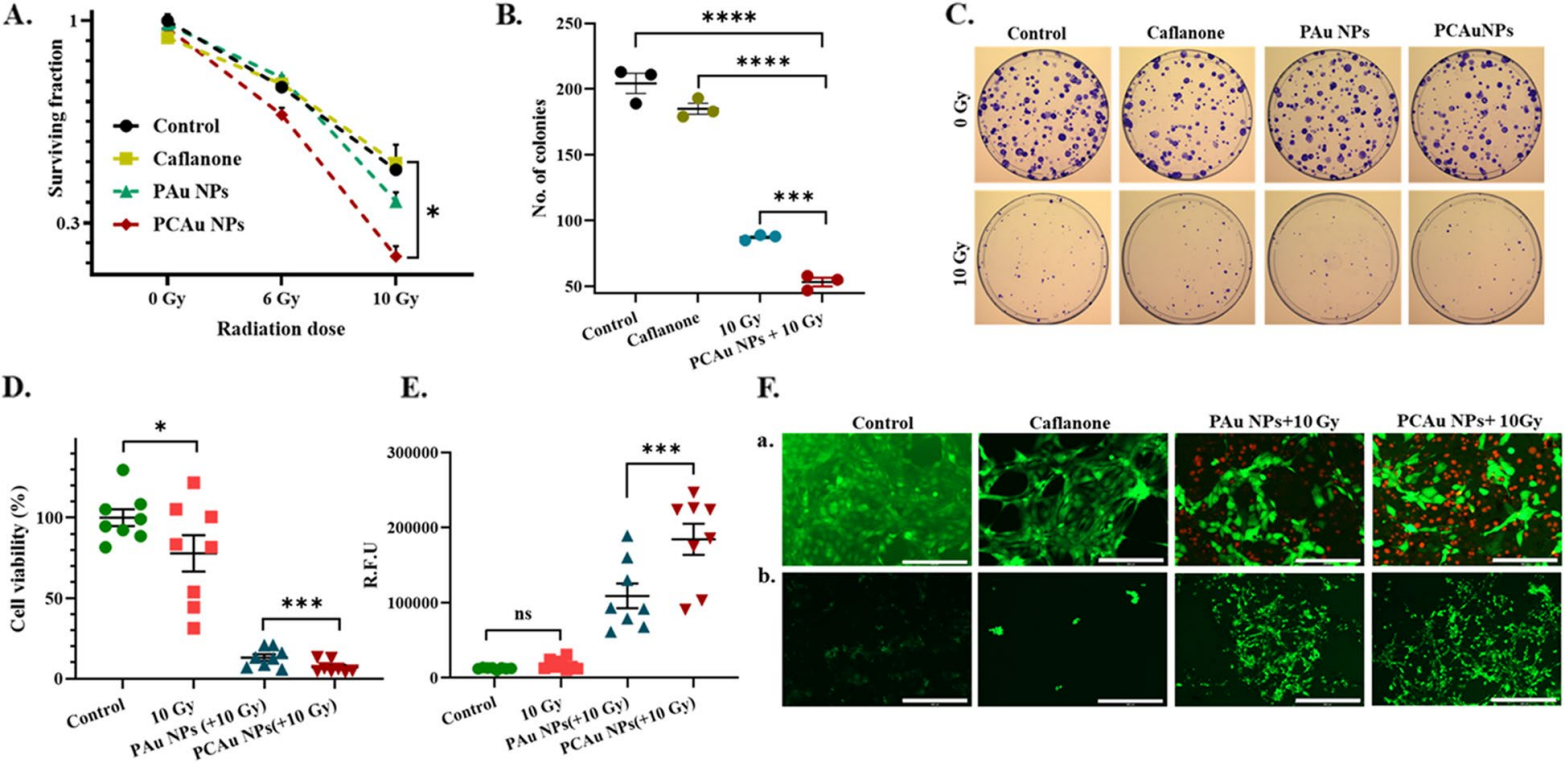
In vitro therapeutic efficacy against pancreatic cancer cells (KPC) **A** Survival fraction of the KPC cells (pancreatic cancer), **B** No. of colonies for each treatment group showing the significant outcome of combinational therapy, **C** Images showing the clonogenic assay (CFU assay), **D** MTT assay showing the significant difference in viability between PAu NPs and PCAu NPs in combination with radiation (10 Gy), **E** DCFHDA assay showing the significant difference in intracellular ROS between blank (PAu NPs) and drug-loaded nanoparticles (PCAu NPs) in combination with radiation (10 Gy), **F** Microscopic images showing a) Live/Dead assay showing the effect of treatment with nanoparticles and radiation (FDA stains live cells in green and PI stains dead cells in red) (*Scale bar corresponds to 200 µm), b). DCFHDA assay showing the intracellular ROS within KPC cells with treatment (*Scale bar corresponds to 400 µm) Statistics: Student t-test; *p* ≤ 0.0001: ****, *p* ≤ 0.001: ***, *p* ≤ 0.01: **, *p* ≤ 0.05: *

The cell viability was also measured by MTT assay to understand if the drug release with radiation had any significant effect. As can be seen from Fig. 5D, a dose-dependent effect was observed, i.e., increased cytotoxicity with increased concentrations of nanoparticles. A closer look at the treatment with PAu NPs (~ 40% viability) and PCAu NPs (~ 13%viability) (Fig. 5E) shows a significant effect on the viability of cells compared to blank nanoparticles, owing to the second line of treatment with radiation mediated release of the drug Caflanone. Hence, a single nanosystem can be used for radio sensitization and drug release, significantly increasing the therapeutic outcome. The cells were also stained (Fig. 5F and Figure S7) with FDA and PI that stained the live and dead cells in green and red, respectively. A majority of dead cells in the group treated with PCAu NPs and radiation (10 Gy), indicated the increased cell death with the combinational treatment approach. We further investigated the effect of treatment on the intracellular ROS. The intracellular ROS increased with the increasing doses of nanoparticle treatment: both PAu NPs and PCAu NPs (Fig. 5G). The difference between blank nanoparticles (PAu NPs) and drug-loaded nanoparticles (PCAu NPs) could also be clearly understood from Fig. 5H, showing a significant increase (~ 1.5 folds) in the intracellular ROS with PCAu NPs(+ 10 Gy) as compared to only PAu NPs (+ 10 Gy). The increased ROS could interact with cellular components like lipids, DNA, and proteins, causing lipid peroxidation protein denaturation and deoxyribonucleic acid damage [2, 10, 22]. The intracellular ROS within the cells (green fluorescence) with different treatment groups (Fig. 5I and Figure S8) shows the cells treated with PCAu NPs and radiation (10 Gy) exhibit bright green fluorescence, indicating the increased intracellular ROS, hinting the beginning of chemical and biological effects following the radiation, leading to cell death [2].

b. Pancreatic cancer (KPC):

The clonogenic survival assay (CFU) was performed to evaluate the combinational therapeutic outcome in pancreatic cancer cells. We tested the effect of the treatment with only radiation, only drug (Caflanone), only nanoparticles (PAu NPs), and Caflanone loaded nanoparticles (PCAu NPs) for different doses of radiation (0, 4, 6, 10 Gy) (Fig. 6A-C and Figure S9). The radiation dose of 10 Gy was noted to be effective (*p* = 0.0163) for curbing pancreatic cancer cell proliferation and the ability to form colonies with the treatment (Fig. 6A). The efficacy of the combinational approach using nanoparticles (PCAu NPs + 10 Gy) can be clearly understood (Fig. 6B) when compared with the individual treatments: only Caflanone i.e., (*p* = 0.0006), only radiation (10 Gy; *p* < 0.0001). Figure 6C shows the number of colonies formed with different treatment groups with and without radiation. The MTT assay (Figure S10) showed a dose-dependent increase in cytotoxicity with the concentration of nanoparticles.

**Fig. 7:**
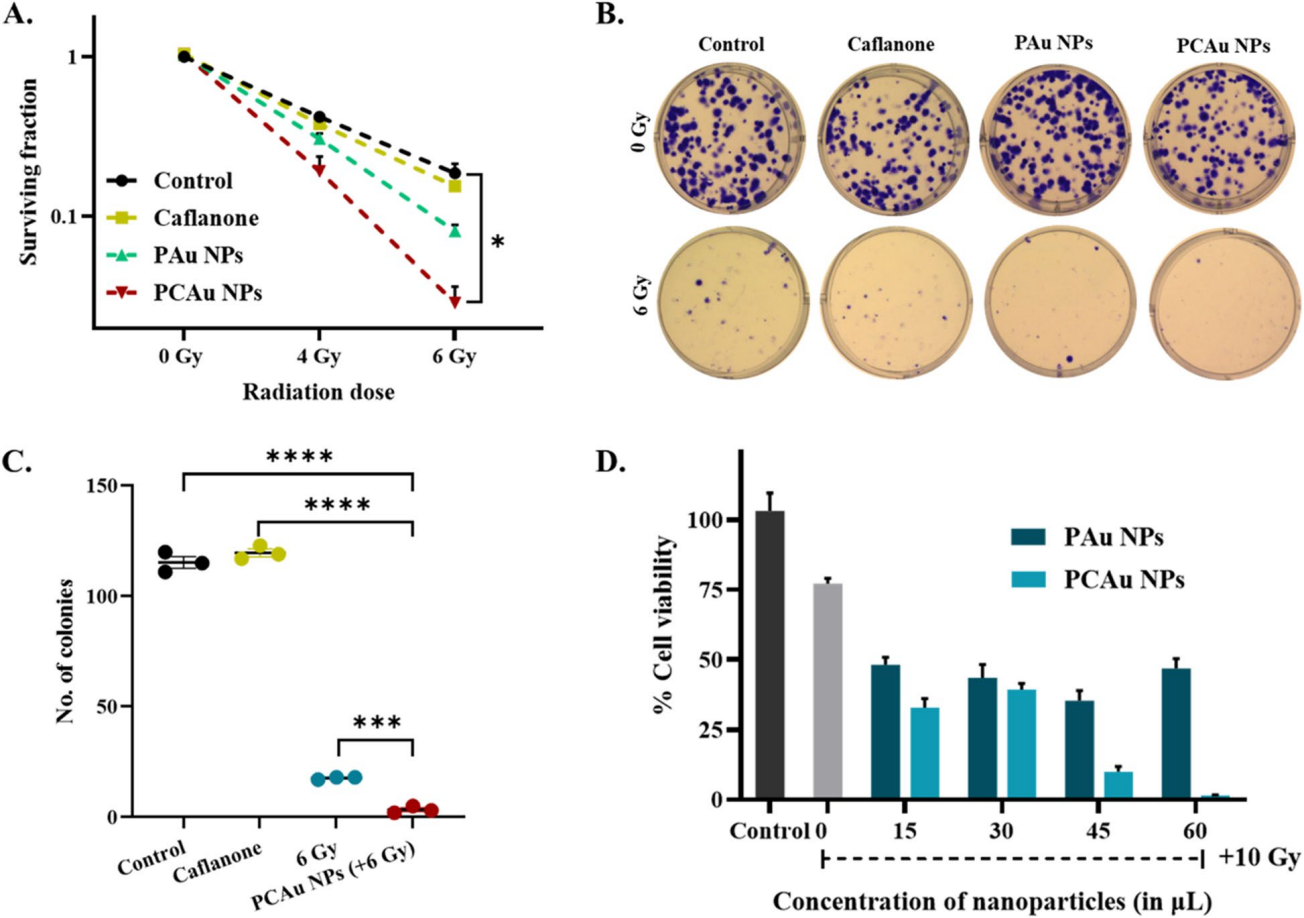
In vitro therapeutic efficacy against glioblastoma (GL261) **A** Survival fraction of the GL261 cells (glioblastoma) with the treatment of drug-loaded nanoparticles and radiation, **B** Images showing the clonogenic assay (CFU assay) or colonies formed with treatment with different nanoparticles and radiation (6 Gy), **C** No. of colonies for each treatment group showing the significant outcome of combinational therapy, **D** MTT assay in GL261 cells showing the dose-dependent viability with nanoparticles and radiation (10 Gy). Statistics: student t-test; *p* ≤ 0.0001: ****, *p* ≤ 0.001: ***, *p* ≤ 0.01: **, *p* ≤ 0.05: *

The MTT assay (Fig. 6D) shows a clear difference in the therapeutic outcome with PCAu NPs as compared to the blank nanoparticles (PAu NPs) and only radiation, bringing out the significance (*p* = 0.0006) of the effect of the drug and the enhanced radiation with the nanosystem. The intracellular ROS (Fig. 6E) also shows the combinational effect of the nanoparticles with a significant increment (*p* = 0.0005) of intracellular ROS in the groups treated with PCAu NPs (~ 1.7 folds) and radiation as compared to only radiation or blank nanoparticles and radiation. The live/dead assay (Fig. 6Fa & Figure S11) shows the microscopic images of the live (in green) and dead cells (in red) with individual treatments and a combinational approach with PCAu NPs triggered with radiation, enforcing the effect of the multifunctional nanoparticles in enhancing the therapeutic outcomes. The intracellular ROS marked by green fluorescence (Fig. 6Fb & Figure S12) also demonstrates a considerable number of cells with increased ROS, indicating the efficacy of the combinational treatment.

c. Glioblastoma (GL261):

The effect of the combinational treatment approach using multifunctional nanoparticles (PCAu NPs) has also been evaluated against glioblastoma cells (GL261). The clonogenic survival (Fig. 7A) shows the effect of different treatments for various doses of radiation (0, 4, and 6 Gy). The PCAu NPs for the two doses of radiation show a significant decline (*p* = 0.0178) in survival compared to all the other groups. The colonies in Fig. 7B (& Figure S13) show the minimal or negligent number of colonies formed with the PCAu NPs compared to radiation or Caflanone alone. A comparison of the number of colonies (Fig. 7C) clearly shows the significant therapeutic outcome with the PCAu NPs, owing to their ability to enhance radiosensitivity and release the drug Caflanone upon irradiation as compared to the individual therapies. A significant decline (*p* ≤ 0.0001) in the number of colonies was found with the combinational treatment as compared to the only drug (~ 37 folds) and only radiation (~ 5.5 folds). Also, dose-dependent cytotoxicity was observed with the concentration of nanoparticles (Fig. 7D).

These in vitro studies in three different cancer cell types establish the nanoparticle's therapeutic efficacy with a combinational approach. From the above results, it can be clearly understood that the multifunctional nanoparticles deliver the drug upon subjecting to radiation, and the significant increase in the intracellular ROS marks the beginning of biological reactions leading to cell death. The increased intracellular ROS could further trigger several reactions within the cells, like lipid peroxidation and DNA damage, eventually leading to cell death. Our future studies will evaluate the mechanism of action of the drugs and treatment and the mode of death to understand the underlying causes and mechanisms of the observed therapeutic efficacy. From the above results, it can be clearly understood that physical therapy, irrespective of the type of cancer, i.e., radiation, currently used for several types of tumors, can be enhanced, and improved by using nanoparticles that can not only be used for radio sensitization but also deliver drugs with radiation. Radiation, the first line of treatment, can be significantly improvised with multifunctional nanoparticles. With these encouraging results in vitro, we further investigated if nanoparticles could be applied for imaging and evaluated their therapeutic efficacy in vivo.

### In vivo studies

**Fig. 8:**
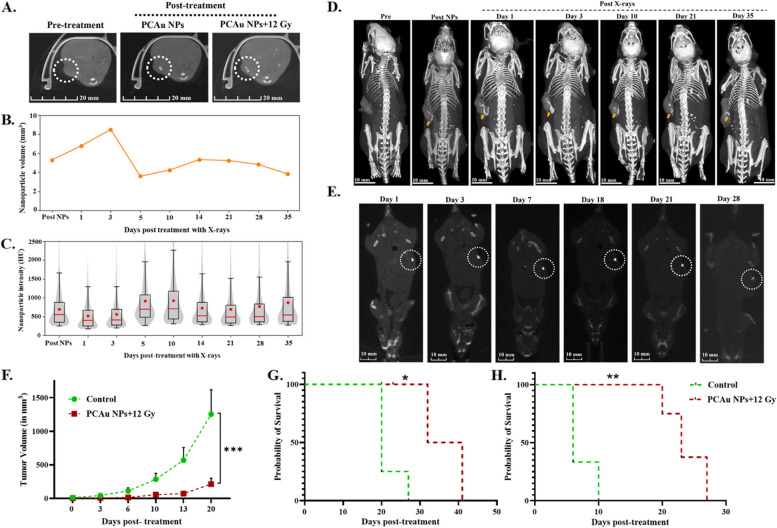
In vivo imaging and therapeutic efficacy **A**. X-ray/CT contrast within the tumor region with the treatment of PCAu NPs and radiation (*Scale bar corresponds to 20 mm), **B**. The nanoparticle volume and **C**. The nanoparticle intensity within the tumor, **D**. Drug-loaded and gold-coated lipo-polymeric hybrid nanoparticles (PCAu NPs) as X-ray/CT contrast agents in vivo in pancreatic tumor model (KPC) (*Scale bar corresponds to 10 mm), **E**. X-ray/CT contrast of PCAu NPs in vivo in breast cancer (4T1) model (*Scale bar corresponds to 10 mm). The effect of combinational treatment on the **F **Tumor volume and **G** Survival in sub-cutaneous breast cancer model (4T1) with one tumor on one flank of the mice. **H** Prolonged survival of the mice bearing subcutaneous breast cancer tumors: metastatic model (two tumors per mouse) treated with combinational treatment. Statistics: Two-way ANOVA followed by Tukey’s test and Log-rank test; *p*  ≤ 0.0001: ****, *p*  ≤ 0.001: ***, *p*  ≤ 0.01: **, *p*  ≤ 0.05: *

- X-ray/CT imaging:

The PCAu NPs showed excellent X-ray/CT contrast in Fig. 4C. We tried to understand if this contrast could also be seen in vivo and how long the nanoparticles could be retained within the tumor. Figure S14 illustrates the CT data processing framework, which includes generating volume and contrast over time, the 3D visualization of the tumors and nanoparticle contrast. The imaging studies were performed in two different tumor models: the pancreatic tumor model (KPC) and the breast cancer model (4T1). A detailed imaging study and analysis was performed using the KPC model. The PCAu NPs were intratumorally injected, and the mice were imaged before and after the injection of nanoparticles (Fig. 8A and Figure S15A). The mice were then subjected to radiation (12 Gy), and following the irradiation, a change in the distribution of nanoparticles was observed as shown in Fig. 8A: post-treatment. To understand this further, we have imaged a group of mice (*n* = 5) before and after the irradiation and continued imaging at regular intervals. Figure 8B shows the distribution of the nanoparticles and the X-ray/CT contrast up to Day35. An increase in the volume of the nanoparticles (Fig. 8B and Figure S15B) and slight differences in the intensity (Fig. 8C& Figure S15C) is noted with irradiation. Post-treatment with X-rays, the volume of the nanoparticles (highlighted by yellow arrow in Fig. 8D) increased and then decreased as the days progressed. We hypothesize this could be due to the disintegration of the PCAu NPs into further smaller gold nanoparticles, as seen in the TEM imaging (Fig. 4D). The nanoparticles disintegrated with radiation, are retained within the tumor, and can slowly be cleared from the body. The clearance of these nanoparticles with radiation will be studied in the future, which can help us establish the biodegradability and clearance of this nanosystem, which are crucial for clinical translation.

b. Safety profile and therapeutic efficacy of the nanoparticles.

We studied the safety profile of PCAu NPs in healthy Balb/C mice. Healthy mice treated with PCAu NPs, were evaluated for body weight, complete blood count, clinical chemistry, and histopathology of major organs for the treatment periods of 3 h, 7 days, 14 days, and 30 days, and were compared to control mice (i.e., non-treated mice). Mice treated with the nanoparticles did not show any significant weight loss, compared to the control mice (Figure S16). The complete blood count and clinical blood chemistry panels assessed for markers of bone marrow toxicity (such as reticulocyte count), renal toxicity (such as blood urea nitrogen (BUN)) and hepatotoxicity (such as ALP) [34] showed no substantial changes with PCAu NPs treatment (Table S1 and S2). The histopathology of vital organs (liver, lungs, spleen, and kidneys) harvested from mice treated with nanoparticles for 3 h, 7 days, 14 days, and 30 days were scored by a pathologist. The analysis showed no signs of toxicity or any variations in their morphology compared to control untreated mice (Table S3-S6). These results indicate the tolerability and safety profile of PCAu NPs. Following the safety profile of PCAu NPs, we further investigated the in vivo therapeutic efficacy in breast cancer bearing mice. The PCAu NPs were intratumorally injected into the Balb/C mice bearing breast tumors (4T1) and were imaged before (Day 1) and after irradiation (up to Day 28). The 2D images shown in Fig. 8E clearly show the X-ray/CT contrast of the nanoparticles within the tumor. Following the radiation (12 Gy), the mice were monitored for their tumor volume and survival and compared to control mice that did not receive any treatment. The treatment with PCAu NPs and radiation (12 Gy) significantly (*p* = 0.0008) reduced the tumor volume as compared to control mice (Fig. 8F). The survival was also found to be increased for two weeks (*p* = 0.0128) with treatment (Fig. 8G).

We have also investigated the therapeutic efficacy in Balb/C mice bearing two subcutaneous breast tumors on two flanks, one representing the primary tumor and the second representing a metastatic tumor. Only one of the tumors was injected with PCAu NPs (as shown in Figure S17A) and subjected to radiation, while the other tumor received no treatment. The mice were monitored for tumor volume of both treated (Figure S17B) and non-treated tumors (Figure S17C) and compared with controls (that received no treatment). The mice treated with PCAu NPs, and radiation showed a considerable decline in tumor volume growth as compared to control mice. A significant increment in survival (*p* = 0.01) has also been observed, as demonstrated in Fig. 8H. The therapeutic effect observed in this model hints at the abscopal effect [60] of the treatment regimen, which needs to be further studied in detail to understand the immunological aspects and effects of the treatment.

Additional experiments are warranted to thoroughly understand the therapeutic efficacy of the combinational treatment approach with multifunctional nanoparticles. In vivo experiments involving all the individual groups will allow us to appreciate the observed results better. The upcoming experiments will also evaluate the underlying mechanisms to understand the immune aspects of the treatment and explore the possibility of immunotherapy. In addition, similar to the in vitro studies, in different cell lines, in vivo therapeutic efficacy will have to be evaluated for different tumor models.

## Conclusions

In summary, we have successfully synthesized multifunctional lipo-polymeric hybrid nanoparticles and demonstrated their ability to enhance radiosensitization in cancer cells, deliver chemotherapeutic agents triggered by radiation, and show the X-ray/CT contrast. A three-in-one approach for cancer therapy has been demonstrated using a multifunctional nanosystem. The therapeutic efficacy of the combinational therapeutic approach has been demonstrated against three diverse types of cancer cells: breast, pancreatic, and glioblastoma. A multifunctional nanosystem is designed and developed for image-guided therapy. Radiation, as a first-line treatment, also triggers the delivery of chemotherapeutics for second-line therapy, is established. The nanoparticles were retained in the tumor for longer durations, showed X-ray/CT contrast, and demonstrated a significant increment in survival compared to control groups. The nanoparticles showed no adverse effects when evaluated for their safety profile in healthy mice. More studies are required to understand if the therapeutic outcome is synergistic by comparing all the control groups in vivo. In addition, the fate of these nanoparticles after radiation will be studied in detail to understand their clearance from the system. A multifunctional lipo-polymeric nanosystem possessing the excellent properties of both lipids and polymers, when coated with the high-Z element gold to enhance radiosensitivity and deliver drugs triggered by radiation and provide X-ray/CT contrast is demonstrated in this report with a series of in vitro and in vivo experiments.

## Supplementary Information

Supplementary Material 1.

## Data Availability

No datasets were generated or analysed during the current study.
