# Supplementary material for: Engineered multifunctional nanoparticles for enhanced radiation therapy: three-in-one approach for cancer treatment
Source: Mol Cancer. 2025 Mar 6;24:68. doi: 10.1186/s12943-025-02266-1 (PMC11883980; doi:10.1186/s12943-025-02266-1)
Supplement: Supplementary file 1 — Supplementary Material 1. [file 12943_2025_2266_MOESM1_ESM.pdf]

Engineered multifunctional nanoparticles for enhanced radiation therapy: Three-in-one approach for cancer treatment

Tejaswini Appidi[1*], Debarghya China[2], George-Răzvan Ștefan [3], Michele Moreau[1], Serena Mao[1], Esteban Velarde[1], Ngeh Toyang[4], Henry Lowe[4], Aravind Kumar Rengan[5], Kai Ding[1], Wilfred Ngwa[1*]

[1]Department of Radiation Oncology and Molecular Radiation Sciences, Johns Hopkins University School of Medicine, Baltimore, Maryland, USA.
[2]Department of Biomedical Engineering, Johns Hopkins University, Baltimore, Maryland, USA.
[3]Carol Davila University of Medicine and Pharmacy, Bucharest, Romania.
[4]Flavocure Biotech Inc., Baltimore, Maryland, USA.
[5]Department of Biomedical engineering, Indian Institute of Technology Hyderabad, Hyderabad, India.

*Correspondence: tappidi1@jh.edu, wngwa1@jh.edu

Supporting information

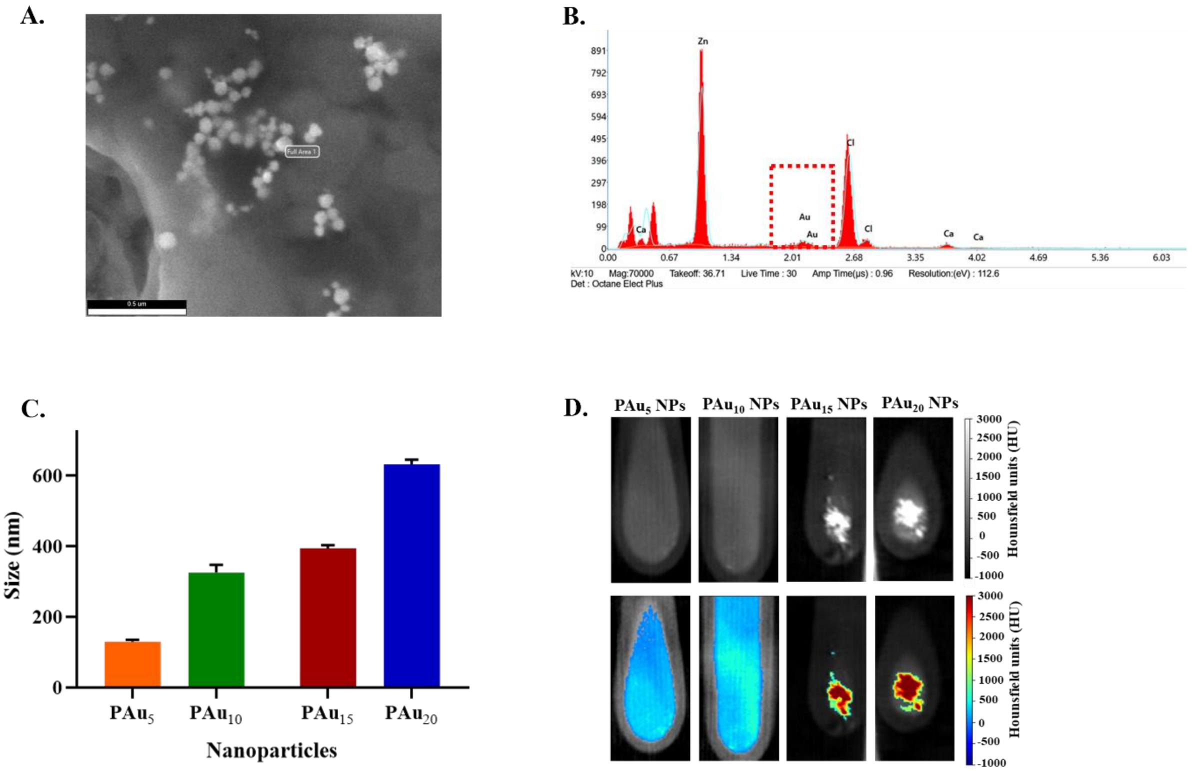

Figure S1: A. SEM analysis of the PAu NPs (*Scale bar corresponds to 0.5µm), B. EDS analysis of the PAu NPs, C. Size of the nanoparticles measured from TEM images, D. Image showing the X-ray/CT contrast of the nanoparticles formed with varying concentrations of gold: Original images matched with the images in color show the intensity.

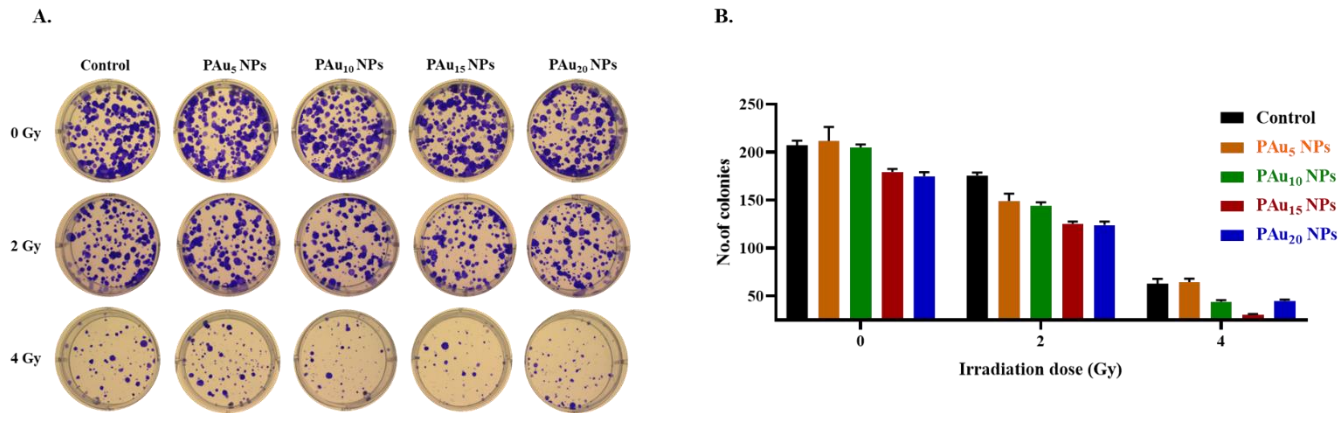

Figure S2: Clonogenic assay in breast cancer cells (4T1). A. Images showing the no. of colonies formed with the treatment of nanoparticles and radiation, B. No. of colonies formed with the treatment of nanoparticles and radiation.

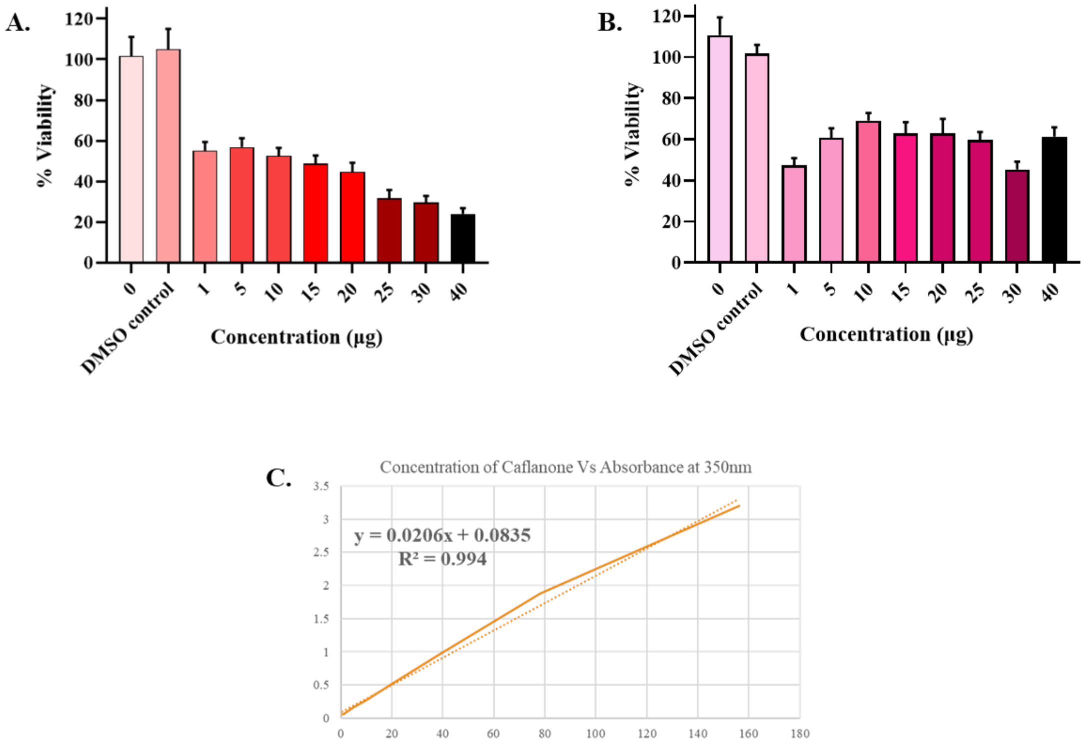

Figure S3: MTT assay showing the effect of Caflanone in A. breast cancer cells (4T1 cells), B. pancreatic cancer cells (KPC cells), C. Concentration curve of Caflanone for evaluation of encapsulation efficiency in nanoparticles.

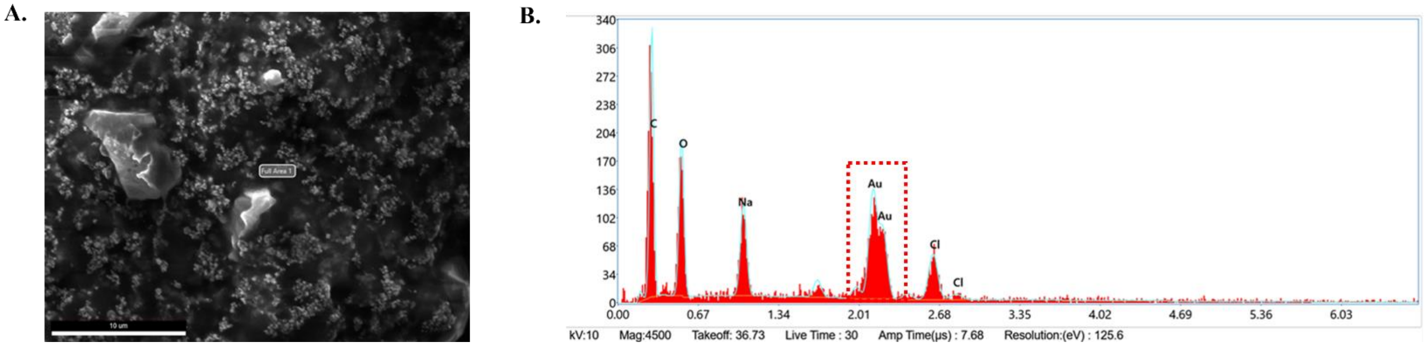

Figure S4: A. SEM image (*Scale bar corresponds to 10μm), and B. EDS analysis of PCAu NPs.

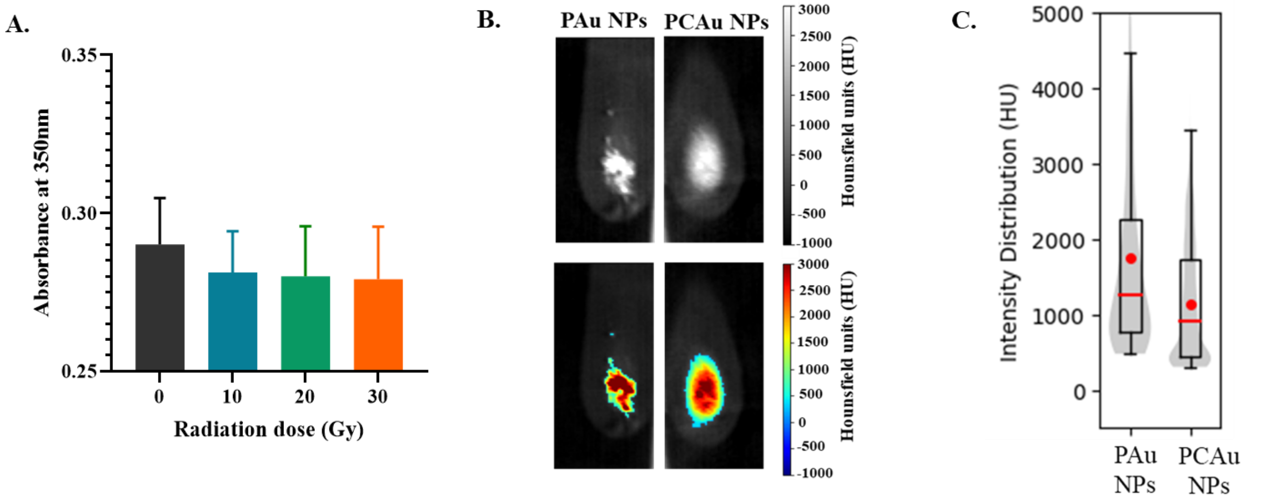

Figure S5: A. Release of Caflanone from PC NPs with radiation, B. Image showing the X-ray/CT contrast of the blank (PAu NPs) and drug-loaded nanoparticles (PCAu NPs): Original images matched with the images in color showing the intensity, C. Quantified X-ray/CT intensity of the nanoparticles.

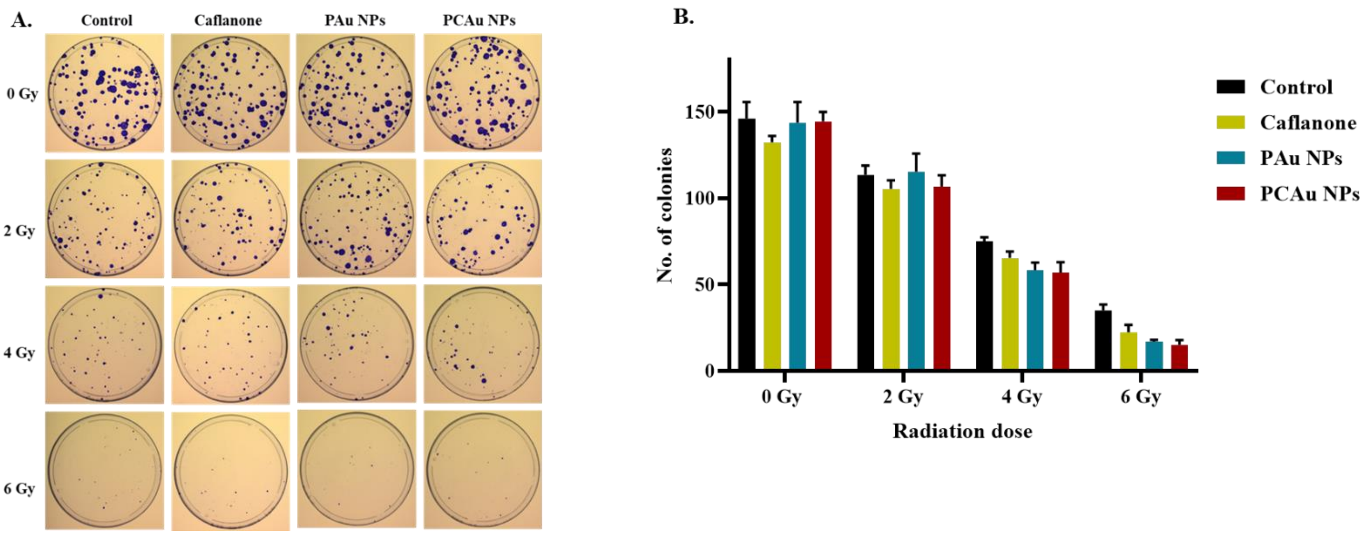

Figure S6: Clonogenic (CFU) assay in breast cancer cells (4T1) showing the number of colonies.

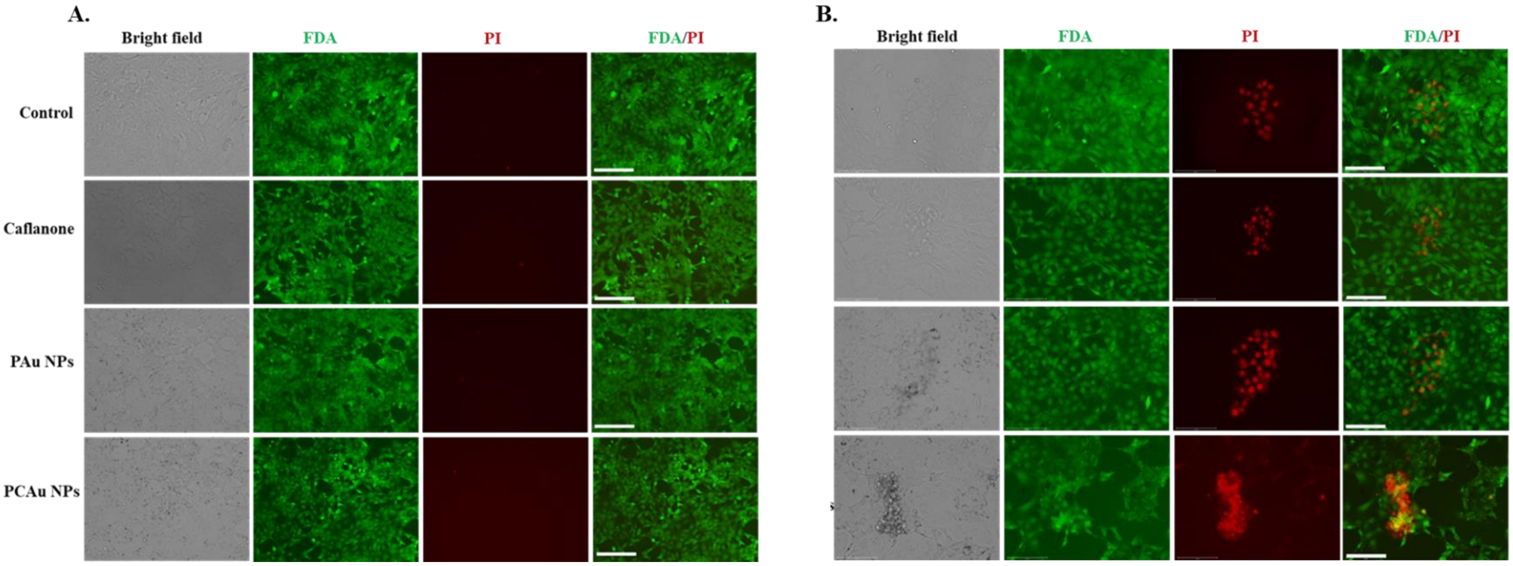

Figure S7: Live/Dead assay in breast cancer cells (4T1). A. 0Gy and B. 10Gy (*Scale bar corresponds to 150μm) FDA stains live cells in green and PI stains dead cells in red.

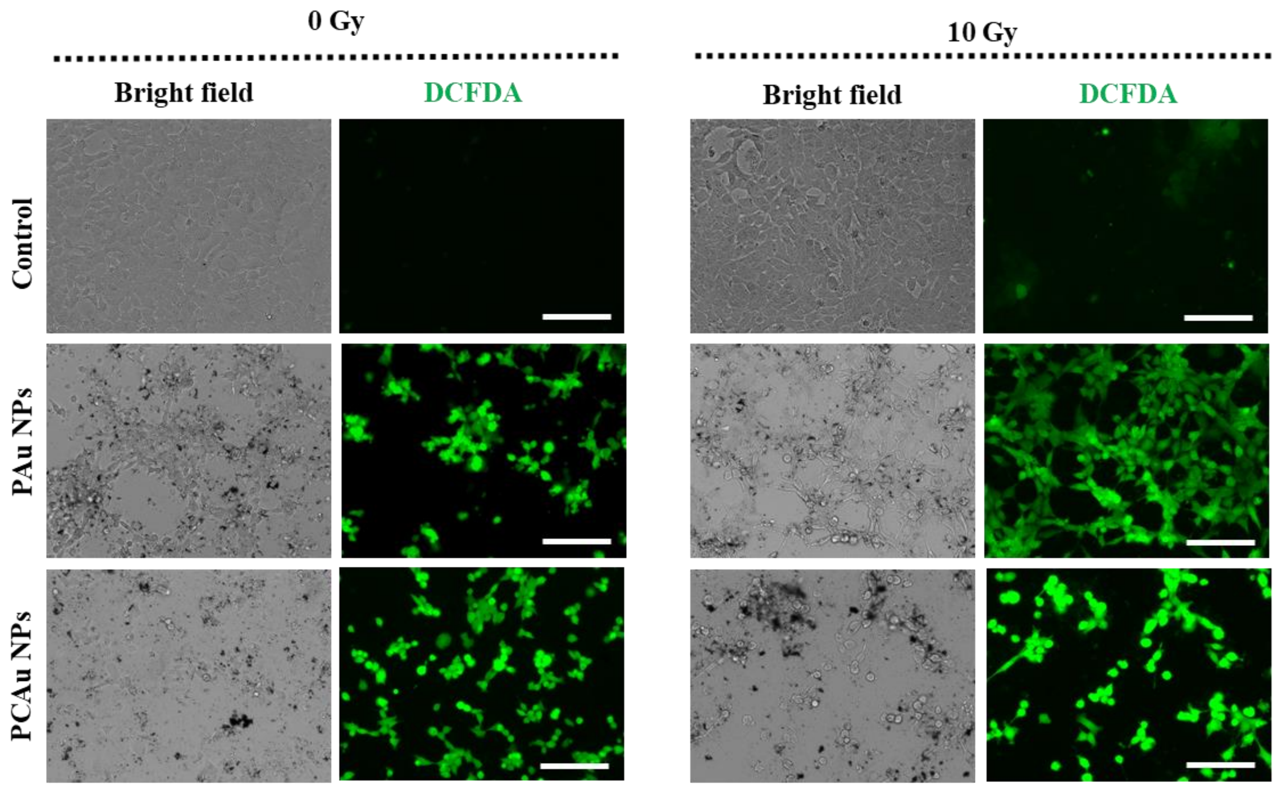

Figure S8: DCFHDA assay in breast cancer cells (4T1) showing the intracellular ROS. (*Scale bar corresponds to 150µm)

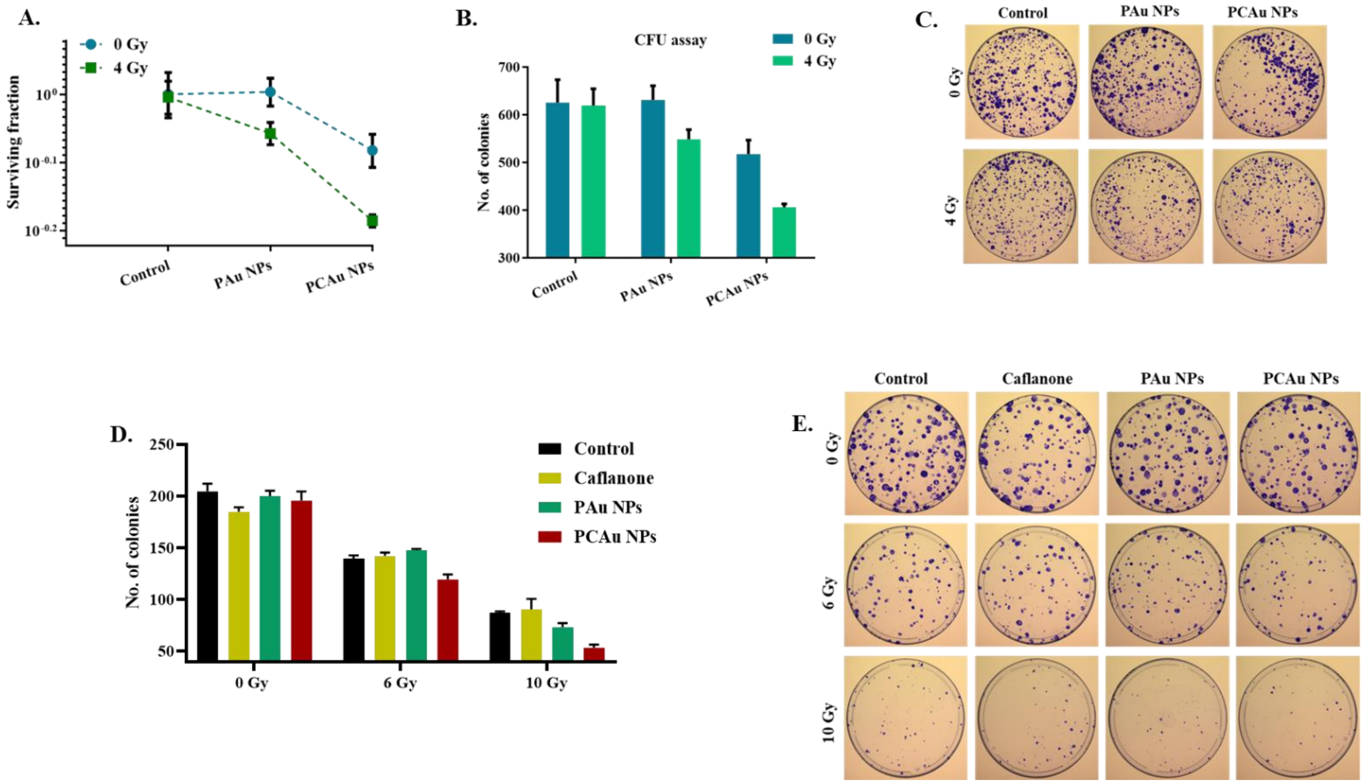

Figure S9: Clonogenic assay in Pancreatic cancer cells (KPC cells): Optimization of radiation dose for treatment

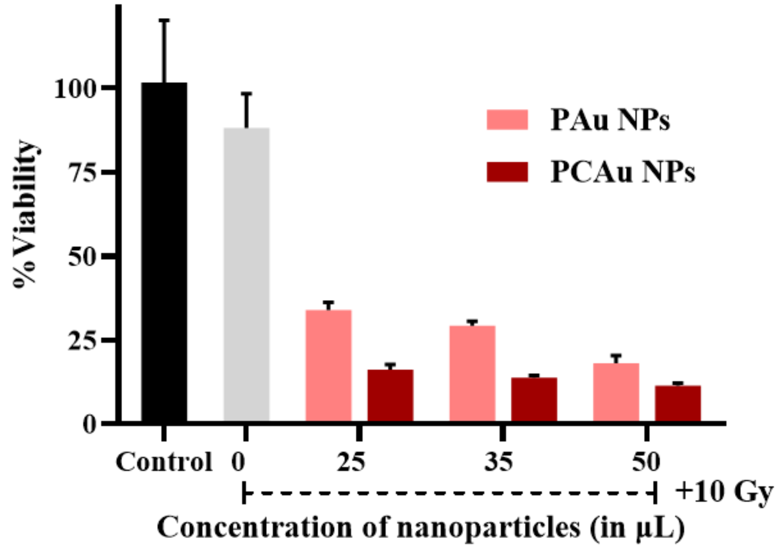

Figure S10: MTT assay in Pancreatic cancer cells (KPC cells) showing the dose-dependent effect of nanoparticles.

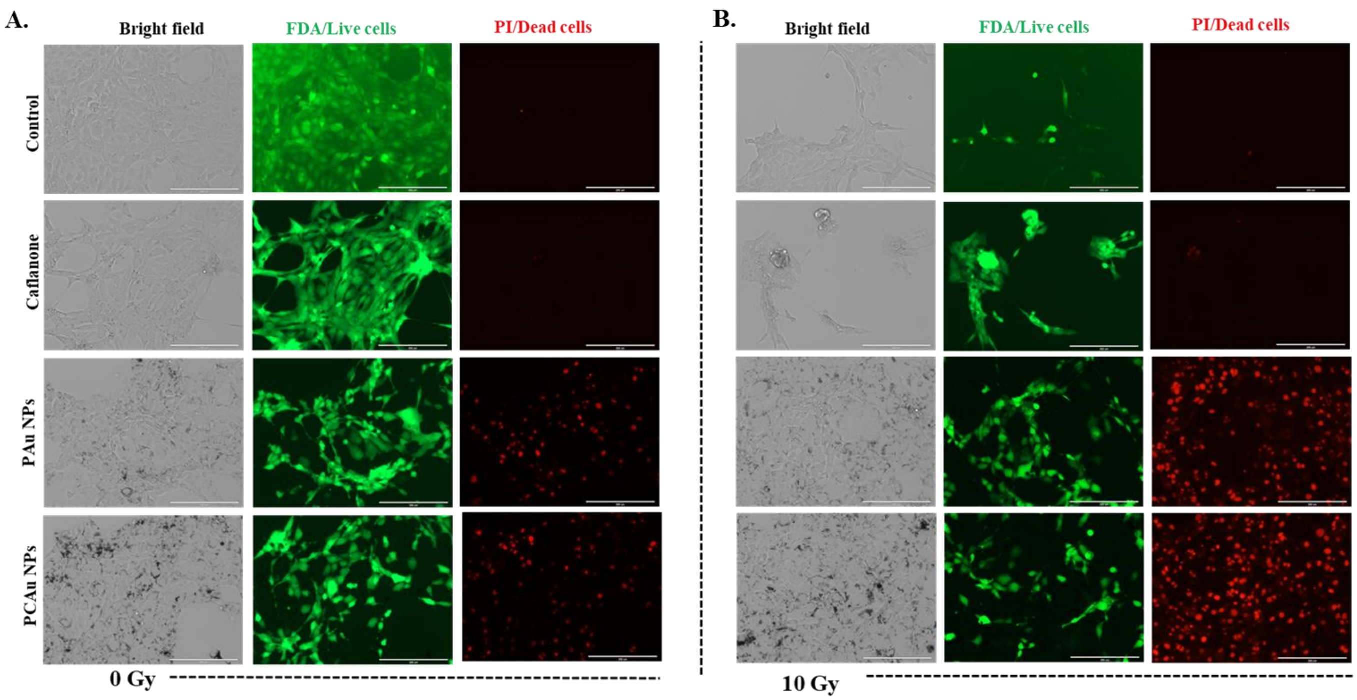

Figure S11: Live/Dead assay in pancreatic cancer cells (KPC). A. 0Gy and B. 10Gy (*Scale bar corresponds to 200µm) FDA stains live cells in green and PI stains dead cells in red.

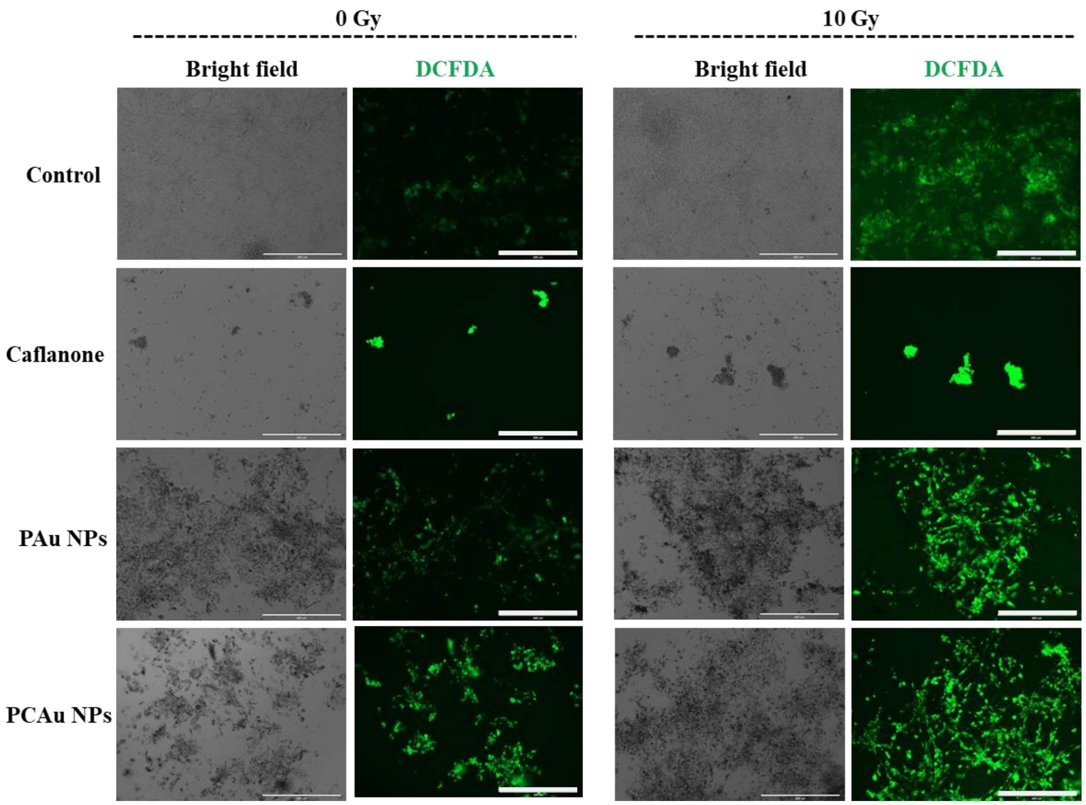

Figure S12: DCFHDA assay in pancreatic cancer cells (KPC) showing the intracellular ROS (*Scale bar corresponds to 400µm)

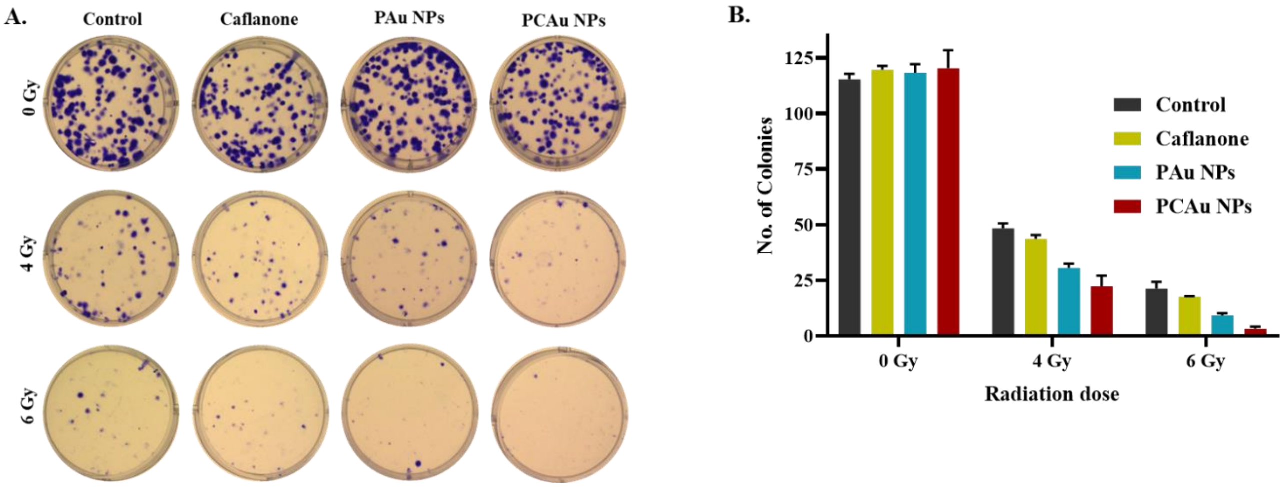

Figure S13: Clonogenic assay (CFU) in glioblastoma cells (GL261).

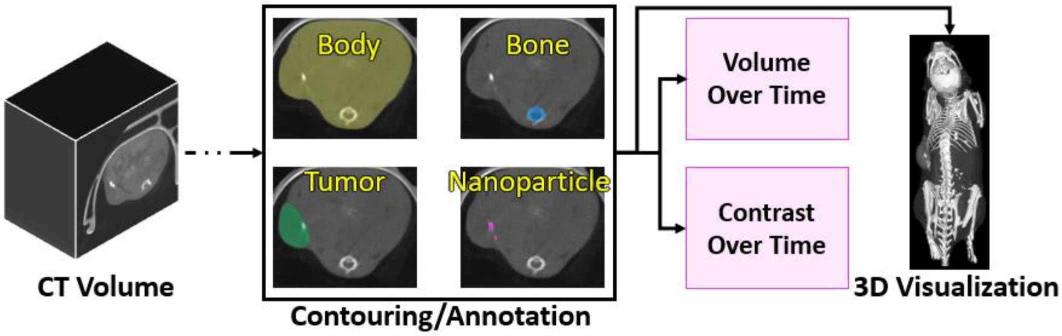

Figure S14: Mice CT volume processing framework.

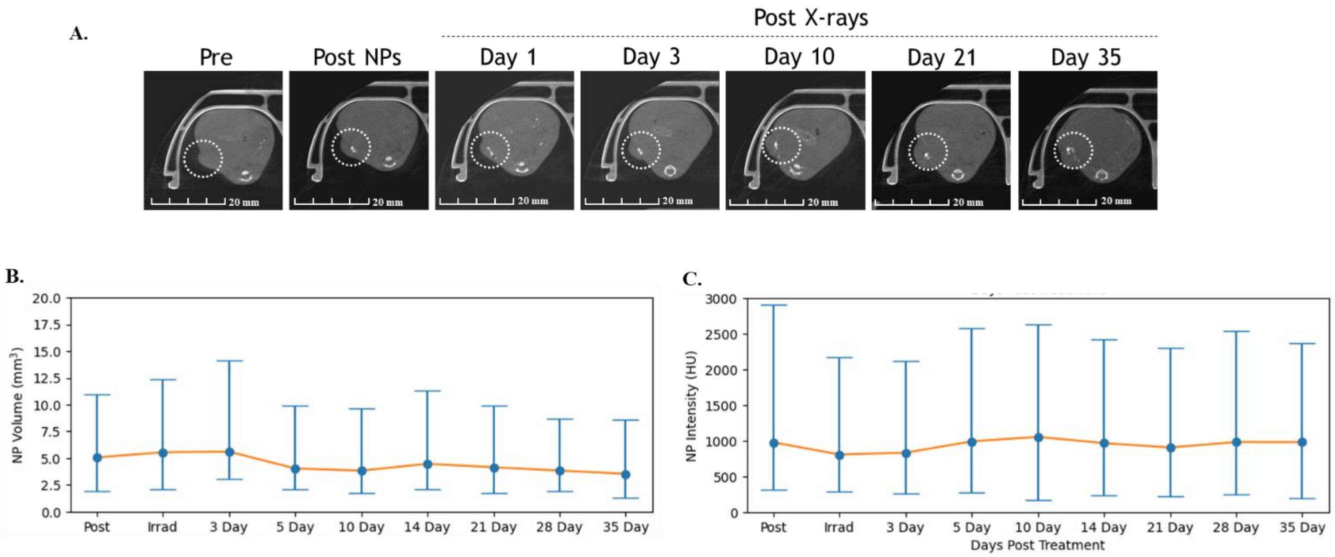

Figure S15: *In vivo* imaging in KPC model. A. The contrast of PCAu NPs within the tumor after treatment with radiation (12 Gy)(*Scale bar corresponds to 20mm), B. Volume and C. Intensity of nanoparticles within the tumor after subjecting to radiation (12 Gy).

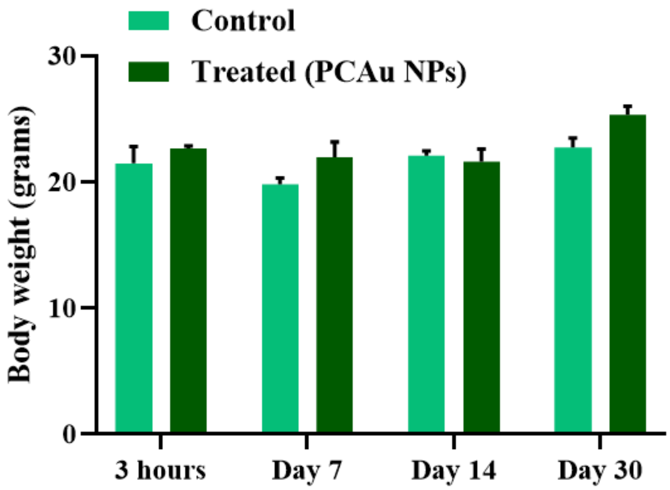

Figure S16: Safety profile of the nanoparticles in healthy Balb/C female mice: Body weight measurement.

Table S1: Safety profile of the nanoparticles: Complete blood count

Parameter	3 hours		Day 7		Day 14		Day 30		Reference range
	Control	Treated	Control	Treated	Control	Treated	Control	Treated	
**WBC**	3.54±0.2	2.06±0.1385	4.20±1.24	3.81±0.68	4.356±0.73	4.81±2.16	2.54	4.425±0.585	3.2-12.7 K/uL
**RBC**	9±0.29	9.38±0.037	8.85±0.29	9.425±0.105	9.544±0.12	8.4275±0.488	9.42	9.52±0.04	7-10.10 M/uL
**HGB**	14.25±0.45	15.43±0.03	14.1±0.32	14.95±0.35	15.46±0.256	13.6±0.813	15.3	15.1±0.1	11.8-14.9 g/dL
**HCT%**	41.6±1.2	44.96±0.23	42.6±1.30	44.35±0.65	45.96±0.857	40.1±2.36	45.5	45.3±0.3	36.7-46.8%
**MCV**	46.2±0.1	47.96±0.12	47.93±0.52	47.05±0.15	48.16±0.434	47.55±0.17	48.3	47.6±0.5	42.2-59.2 fL
**MCH**	15.9	16.433±0.03	15.86±0.12	15.8±0.2	16.18±0.139	16.15±0.13	16.3	15.9±0.2	13.8-18.4 pg
**MCHC**	34.35±0.05	34.33±0.145	33.06±0.33	33.6±0.3	33.62±0.308	33.925±0.32	33.7	33.4±0	31.0-34.7 g/dL
**CHCM**	31.4±0.5	31.366±0.088	30.9±0.30	31.3±0.5	30.5±0.27	31±0.21	30.3	30.6±0.1	
**CH**	14.5±0.3	15.03±0.03	14.8±0.1	14.7±0.3	14.7±0.05	14.725±0.08	14.6	14.55±0.15	
**RDW%**	13.05±0.05	13.13±0.23	13.23±0.13	13.85±0.25	13.38±0.066	13.55±0.132	13.3	13.2	11.7-15.1%
**HDW**	2.15±0.02	2.14±0.03	2.086±0.01	2.215±0.075	2.164±0.026	2.1975±0.02	2.12	2.13	
**PLT**	320±118	595.66±109.34	696.33±191.38	438.5±141.5	543.6±73.66	410.25±224.11	949	607.5±103.5	786-1657
**MPV**	13.6±2.8	8.2±0.65	7.13±0.28	10.55±0.45	8.18±0.58	14.55±4.58	7	7.05±0.55	
**NEUT %**	19.4±0.1	30.13±3.33	12.13±0.56	25.75±0.05	14.44±2.41	12.35±1.211	15	11.1±5.9	
**NEUT Count**	0.685±0.045	0.61±0.03	0.52±0.17	0.985±0.175	0.64±0.13	0.65±0.30	0.38	0.525±0.325	0.5-2 K/uL
**Lymph %**	74.5±0.9	51.2±4.99	65.06±16.75	66.4±0.8	75.4±3.44	81.8±2.15	77.6	78.9±1.5	
**Lymph count**	2.635±0.185	1.063±0.169	3.433±1.01	2.54±0.48	3.25±0.53	3.88±1.766	1.97	3.48±0.4	3.8-8.9 K/uL
**Mono%**	2.3±0.5	7.433±0.46	1.6±0.05	2.8±0.2	2.38±0.37	1.575±0.06	1.4	2.35±0.75	
**Mono count**	0.08±0.01	0.153±0.02	0.066±0.02	0.11±0.01	0.102±0.019	0.075±0.03	0.03	0.1±0.02	0.0-0.3 K/uL
**EOS%**	3.45±0.45	10.96±2.34	4.1±0.86	4.6±0.7	7.5±2.33	3.925±1.115	5.4	7.35±3.75	
**EOS count**	0.12±0.01	0.21±0.035	0.15±0.029	0.17	0.35±0.155	0.1875±0.099	0.14	0.305±0.125	0.00-0.40 K/uL
**BASO %**	0.1	0.06±0.03	0.06±0.03	0.1±0.1	0.12±0.05	0.15±0.086	0.2	0.1	
**Baso count**	0.005±0.005	0	0.003±0.0033	0.005±0.005	0.006±0.002	0.005±0.002	0.01	0	0-0.1 K/uL
**LUC %**	0.25±0.05	0.26±0.088	0.33±0.088	0.35±0.05	0.12±0.037	0.2±0.04	0.4	0.2±0.1	
**LUC count**	0.01	0.003±0.003	0.02±0.01	0.015±0.005	0.006±0.002	0.01±0.007	0.01	0.005±0.005	
**Retics %**	2.33±0.21	3.27±0.43	3.073±0.217	4.32±0.08	3.374±0.234	3.005±0.21	2.81	2.665±0.465	

*****WBC: white blood cells; RBC: Red blood cells; HGB: hemoglobin; HCT: Hematocrit; MCV: Mean corpuscular volume; MCH: Mean corpuscular hemoglobin; MCHC: Mean corpuscular hemoglobin concentration; CHCM: cellular hemoglobin concentration

mean; CH: congenital hypothyroidism; RDW: Red cell distribution width; HDW: hemoglobin distribution width; PLT: platelet count; MPV: mean platelet volume; NEUT: neutrophil count; EOS: eosinophil; BASO: basophil; LUC: large unstained cells.

Table S2: Safety profile of the nanoparticles: clinical chemistry

Parameter	3 hours		Day 7		Day 14		Day 30		Reference range
	Control	Treated	Control	Treated	Control	Treated	Control	Treated	
**ALB/GLOB Ratio**	1.35±0.05	1.7±0.1	1.4±0.2	1.35±0.05	1.55±0.15	1.43±0.03	1.7±0.2	2.25±0.85	7-10.10 M/uL
**Albumin**	2.75±0.15	3	2.85±0.05	2.85±0.05	2.95±0.05	2.9±0.05	3.05±0.05	2.8	11.8-14.9 g/dL
**Alkaline phosphatase**	56.5±1.5	73.5±4.5	66±4	58±1	66±2	80.66±4.70	58±22	73±3	36.7-46.8 %
**ALT**	30.5±6.5	37.5±10.5	33±7	65±21	64.5±0.5	48±5.6	215±164	40±6	42.2-59.2 fL
**AST**	62±13	75.5±18.5	52±8	91.5±19.5	96.5±0.5	68±3.5	228±136	61±6	13.8-18.4 pg
**BUN**	18.5±1.5	20.5±1.5	24.5±2.5	17.5±1.5	18.5±0.5	17.66±0.88	20.5±0.5	17±1	31.0-34.7 g/dL
**Creatinine**	0.085±0.015	0.105±0.005	0.11±0.01	0.275±0.175	0.12±0.01	0.3±0.11	0.21±0.13	0.105±0.015	
**Bun/Creatinine ratio**	228±58	196.5±23.5	222.5±2.5	113±77	155.5±17.5	97±51.5	161±102	166.5±33.5	
**Globulin**	2.05±0.05	1.8±0.1	2.05±0.25	2.1	1.95±0.15	2±0.05	1.85±0.25	1.45±0.55	11.7-15.1 %
**Glucose**	186.5±39.5	170.5±0.5	238±23	172.5±34.5	176.5±4.5	219.66±6.17	171.5±47.5	173±18	
**Phosphorus**	9.75±2.15	10.85±0.75	8.25±0.95	10.25±1.95	9.55±0.35	9.53±0.66	9.05±2.75	8.3±0.6	786-1657
**Sodium**	150.6±2.8	147.15±2.15	150.05±1.25	150.4±1.7	149.3±2.9	149.03±1.30	145.5±1.6	144.35±1.55	
**Potassium**	6.24±0.31	5.215±0.155	6.485±0.205	6.575±0.145	6.095±0.835	6.12±0.52	6.605±0.235	6.695±0.145	
**Na/K ratio**	24±1	28.5±0.5	23±1	23±1	25±3	24.66±2.02	22.5±0.5	21.5±0.5	0.5-2 K/uL
**Bilirubin direct**	0.35±0.15	0.45±0.05	0.25±0.05	0.4±0.1	0.5±0.1	0.2±0.05	0.6±0.3	0.3	
**Bilirubin indirect**	0.15±0.05	0.25±0.05	0.1±0.1	0.15±0.05	0.1	0.13±0.03	0.1±0.1	0.1	3.8-8.9 K/uL
**Total bilirubin**	0.5±0.2	0.7±0.1	0.35±0.15	0.55±0.15	0.6±0.1	0.33±0.03	0.7±0.4	0.4	

**Total protein**	4.8±0.2	4.8±0.1	4.9±0.2	4.95±0.05	4.9±0.1	4.9±0.1	4.9±0.3	4.25±0.55	0.0-0.3 K/uL

*ALB/GLOB: albumin/globulin; ALT: Alanine aminotransferase; AST: Aspartate aminotransferase; BUN: Blood urea nitrogen; Na/K: sodium-potassium.

Table S3: The histopathology report of the mice treated with PCAu NPs (G_1,2,3) for 3hours (D3Hr) compared to control mice that received no treatment (G_A, B, C).

Mouse Accession Number	24012385	24012386	24012387	24012391	24012392	24012393
Mouse ID or Rx Group	G_A_#1_3Hr	G_A_#2_3Hr	G_A_#3_3Hr	G_1_#1_3Hr	G_1_#2_3Hr	G_1_#3_3Hr
	Control group			Treatment group		
**LUNG - The section shows multiple anatomically normal lobes that are approximately 50% inflated. There are occasional small foci of hemorrhage in the normal lung reportedly resulting from the euthanasia procedure.**	N	N	N	N	N	N
**SPLEEN - The spleen is architecturally correct, from a normal immunocompetent mouse strain with a 4:1 ratio of red pulp to white pulp. The red pulp contains robust extramedullary hematopoiesis, and the white pulp consists of numerous lymphoid follicles.**	N	N	N	N	N	N
**LIVER - There are two sections of liver lobe collected from non-fasted animals, that are anatomically normal.**						N
Perivascular lymphocytic infiltrates				1MF		
Microabscesses - Small aggregates of inflammatory cells, up to 100 cells, consisting of macrophages, lymphocytes, and neutrophils that often contain one or more entrapped degenerating hepatocytes. In microabscesses the neutrophils are the predominate inflammatory cell in the cellular aggregate.					1F	
Microgranulomas - Small aggregates of inflammatory cells, up to 100 cells, consisting of macrophages, lymphocytes, and neutrophils that often contain one or more entrapped degenerating hepatocytes. In microgranulomas the mononuclear inflammatory cells predominate the cellular aggregate.	1MF	1F	2MF			
**KIDNEYS (LEFT AND RIGHT) - The kidneys are anatomically normal.**		N	N	N	n	N
Glomerular degeneration & mineralization	2FU					

Scoring Definitions

0= No finding
1= Minimal
2= Mild
3= Moderate
4= Marked
5= Severe

N= Normal
M= Missing
B=Bilateral
MF=Multifocal
F=Focal
D=Diffuse
U=Unilateral

Table S4: The histopathology report of the mice treated with PCAu NPs (G_1,2,3) for 7 days (D7) compared to control mice that received no treatment (G_A, B, C).

Mouse Accession Number	24012513	24012514	24012515	24012519	24012520	24012521
Mouse ID or Rx Group	G_A_#1_D7	G_A_#2_D7	G_A_#3_D7	G_1_#1_D7	G_1_#2_D7	G_1_#3_D7
	Control group			Treatment group		
**LUNG - The section shows multiple anatomically normal lobes that are approximately 50% inflated. There are occasional small foci of hemorrhage in the normal lung reportedly resulting from the euthanasia procedure.**	N	N	N	N	N	N
Peribronchiolar lymphocytic aggregates						
**SPLEEN - The spleen is architecturally correct, from a normal immunocompetent mouse strain with a 4:1 ratio of red pulp to white pulp. The red pulp contains robust extramedullary hematopoiesis, and the white pulp consists of numerous lymphoid follicles.**	N	N	N	N	N	N
Lymphocytic hyperplasia, white pulp						
**LIVER - There are two sections of liver lobe collected from non-fasted animals, that are anatomically normal.**	N	N			N	N
Perivascular lymphocytic infiltrates			1MF			
Microgranulomas - Small aggregates of inflammatory cells, up to 100 cells, consisting of macrophages, lymphocytes, and neutrophils that often contain one or more entrapped degenerating hepatocytes. In microgranulomas the mononuclear inflammatory cells predominate the cellular aggregate.				1MF		
**KIDNEYS (LEFT AND RIGHT) - The kidneys are anatomically normal. Approximately half the slides contain a section of anatomically normal adrenal gland**	N		N	N	N	N
Infarction - A radiating area of tubular and interstitial renal parenchymal degeneration extending through the full thickness of the cortex from the capsule to the corticomedullary junction, consisting of dilated tubules, interstitial lymphocytic infiltrates, and scenescent glomeruli.		2FU				

Scoring Definitions

0= No finding	N= Normal
1= Minimal	M= Missing
2= Mild	B=Bilateral
3= Moderate	MF=Multifocal
4= Marked	F=Focal
5= Severe	D=Diffuse
	U=Unilateral

Table S5: The histopathology report of the mice treated with PCAu NPs (G_1,2,3) for 14 days (D14) compared to control mice that received no treatment (G_A, B, C).

Mouse Accession Number<br>Mouse ID or Rx Group	24011930<br>G_A_#1_D14	24011931<br>G_A_#2_D14	24011932<br>G_A_#3_D14	24011936<br>G_1_#1_D14	24011937<br>G_1_#2_D14	24011938<br>G_1_#3_D14
	Control group			Treatment group		
**LUNG - The section shows multiple anatomically normal lobes that are approximately 50% inflated. There are occasional small foci of hemorrhage in the normal lung reportedly resulting from the euthanasia procedure.**	**N**	**N**	**N**	**N**	**N**	**N**
**SPLEEN - The spleen is architecturally correct, from a normal immunocompetent mouse strain with a 4:1 ratio of red pulp to white pulp. The red pulp contains robust extramedullary hematopoiesis, and the white pulp consists of numerous lymphoid follicles.**	**N**	**N**	**N**	**N**	**N**	**N**
**LIVER - There are two sections of liver lobe collected from non-fasted animals, that are anatomically normal.**	**N**	**N**	**N**	**N**	**N**	**N**
Perivascular lymphocytic infiltrates						
Microgranulomas - Small aggregates of inflammatory cells, up to 100 cells, consisting of macrophages, lymphocytes, and neutrophils that often contain one or more entrapped degenerating hepatocytes. In microgranulomas the mononuclear inflammatory cells predominate the cellular aggregate.						
**KIDNEYS (LEFT AND RIGHT) - The kidneys are anatomically normal. There is also a section**	**N**	**N**	**N**	**N**	**N**	**N**
**of normal adrenal gland in each kidney section**						

Scoring Definitions

0= No finding
1= Minimal
2= Mild
3= Moderate
4= Marked
5= Severe

N= Normal
M= Missing
B=Bilateral
MF=Multifocal
F=Focal
D=Diffuse
U=Unilateral

Table S6: The histopathology report of the mice treated with PCAu NPs (G_1,2,3) for 30 days (D30) compared to control mice that received no treatment (G_A, B, C).

Mouse Accession Number	24013074	24013075	24013076	24013080	24013081	24013082
Mouse ID or Rx Group	G_A_#1_D30	G_A_#2_D30	G_A_#3_D30	G_1_#1_D30	G_1_#2_D30	G_1_#3_D30
**LUNG - The section shows multiple anatomically normal lobes that are often less than 50% inflated. There are occasional small foci of hemorrhage in the normal lung reportedly resulting from the euthanasia procedure.**	N	N	N	N	N	N
**SPLEEN - The spleen is architecturally correct, from a normal immunocompetent mouse strain with a 4:1 ratio of red pulp to white pulp. The red pulp contains robust extramedullary hematopoiesis, and the white pulp consists of numerous lymphoid follicles.**	N	N	N	N	N	N
**LIVER - There are two sections of liver lobe collected from non-fasted animals, that are anatomically normal.**					N	N
Perivascular lymphocytic infiltrates						
Microgranulomas - Small aggregates of inflammatory cells, up to 100 cells, consisting of macrophages, lymphocytes, and neutrophils that often contain one or more entrapped degenerating hepatocytes. In microgranulomas the mononuclear inflammatory cells predominate the cellular aggregate.	1MF	1MF	1F	1MF		
**KIDNEYS (LEFT AND RIGHT) - The kidneys are anatomically normal. There is also a section of anatomically normal adrenal gland in approximately half of the kidney sections**	N	N	N	N	N	N
Pelvic suburothelial lymphocytic and neutrophilic infiltrates						

Scoring Definitions

0= No finding
1= Minimal
2= Mild
3= Moderate
4= Marked
5= Severe

N= Normal
M= Missing
B=Bilateral
MF=Multifocal
F=Focal
D=Diffuse
U=Unilateral

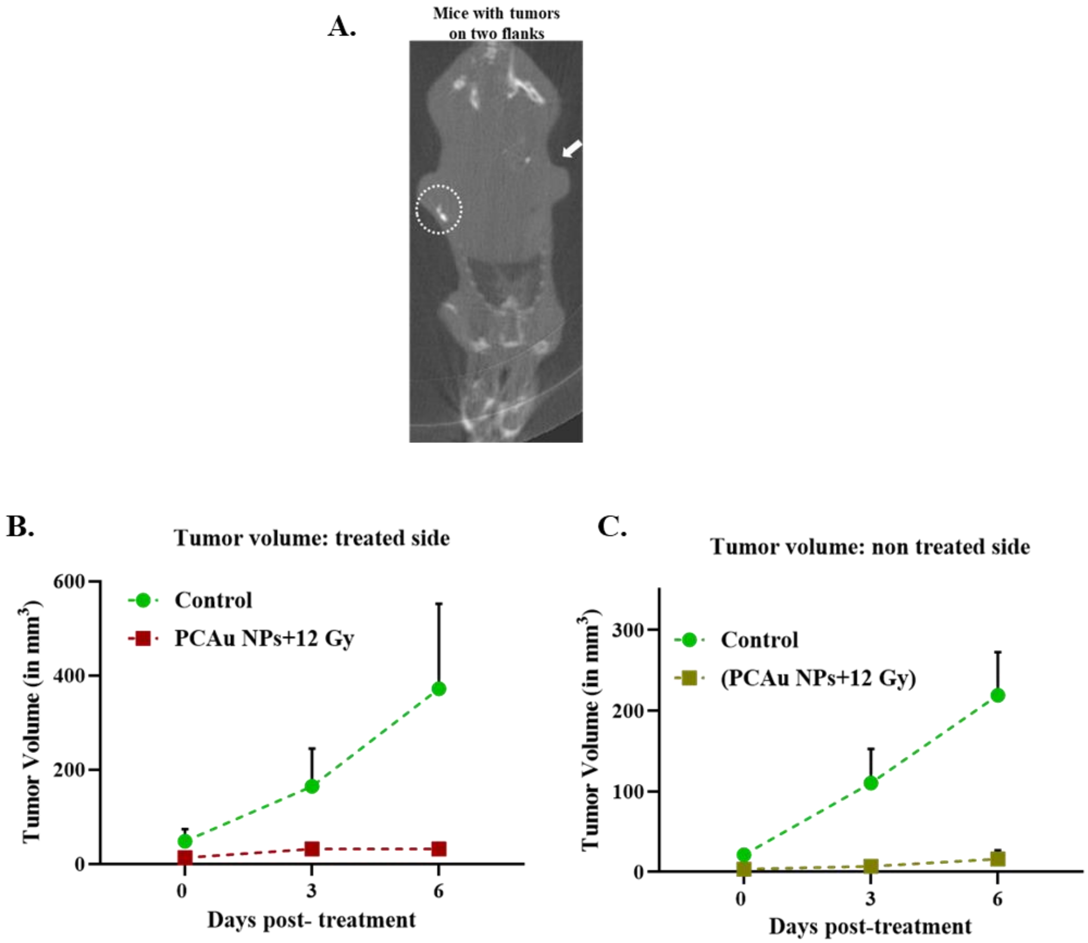

Figure S17: *In vivo* therapeutic efficacy in breast cancer model (4T1). A. CT Image showing two tumors: One on the left side is treated with nanoparticles and radiation (12 Gy), and the one on the right is non-treated side pointed by an arrow, B. The volume of the tumor treated with nanoparticles and radiation (12 Gy), C. The volume of the non-treated side tumor.
